# Tri-domain prediction and optimization of nanocomposite polymers for high-performance 3D printing

**DOI:** 10.1038/s41598-026-43774-4

**Published:** 2026-03-31

**Authors:** Natrayan Lakshmaiya, P. Hari Chandra Prasad, Prabhu Paramasivam, Ramya Maranan, Anand Rajendran, Gemeda Bekele Bacha

**Affiliations:** 1 Department of Research and Innovation, Saveetha School of Engineering, SIMATS, Chennai, Tamil Nadu 602105 India; 2 Department of Mechanical Engineering, Aditya University, Surampalem, Andhra Pradesh 533437 India; 3 Centre for Research Impact & Outcome, Chitkara University Institute of Engineering and Technology, Chitkara University, Rajpura, 140401 Punjab India; 4 Division of Research and Development, Lovely Professional University, Jalandhar - Delhi G.T.Road, Phagwara, Punjab 144411 India; 5 Lloyd Institute of Engineering & Technology, Plot No. 3, Knowledge Park II , Greater Noida, Uttar Pradesh 201306 India; 6 Department of Civil Engineering, Salale University, 245 Salale, Fiche, Ethiopia

**Keywords:** Additive manufacturing, Multi-task attention ensemble, Nanocomposite polymers, Physics-guided AI, 3D printing, Engineering, Materials science, Mathematics and computing

## Abstract

Additive manufacturing (AM) of polymer nanocomposites offers vast potential for creating multi-functional materials with enhanced mechanical, thermal, and electrical properties. However, the nonlinear interdependencies among nanofiller composition, printing conditions, and final performance metrics make traditional empirical optimization inefficient and unpredictable. To overcome these limitations, this study proposes a Physics-Guided Multi-Task Attention Ensemble (PG-MTAE) model that simultaneously predicts and optimizes the tri-domain properties of nanocomposite-enhanced polymers fabricated via Fused Deposition Modeling (FDM). The model integrates material- and process-level descriptors, a feature interaction module for inter-domain dependency learning, and physics-based constraints to ensure physically consistent predictions aligned with established percolation and reinforcement laws. Two benchmark datasets—the Nanocomposites Properties Database and the FDM 3D Printed Composite Material Dataset were harmonized for multi-domain modeling. The implementation was carried out in Python 3.10. Explainability was achieved using SHAP analysis, while Bayesian Optimization was employed to discover Pareto-optimal configurations for maximizing performance trade-offs. The proposed PG-MTAE achieved a determination coefficient (R^2^) of 0.9897, RMSE of 0.0348, and MAE of 0.0219, outperforming traditional ANN, RNN, and hybrid XGBoost models by jointly predicts mechanical, thermal, and electrical properties. Results confirm the model’s ability to capture physics-consistent nonlinear interactions among filler concentration, print energy density, and process temperature. This AI-driven approach provides a virtual material design framework, significantly reducing experimental trial-and-error cycles while enhancing design reliability for high-performance, multifunctional nanocomposites in aerospace, biomedical, and electronic applications.

## Introduction

Additive manufacturing (AM), also referred to as 3D printing, has become a disruptive technology in the next-generation manufacturing because of its design freedom nature, less material waste, and ability to achieve complex and tailored geometries^1^. Polymer-based filaments are still very common against the different AM materials due to their low cost, ease of processing, and adjustable physical characteristics^2,3^. Nevertheless, traditional polymer filaments have a low mechanical strength, low thermal stability, and low electrical conductivity which limits their applications in structural, thermal and electronics applications^4^. To address these shortcomings, researchers have focused more and more on polymer nanocomposites that are reinforced using nanoscale fillers that include carbon nanotubes (CNTs), graphene, silicon carbide (SiC), and alumina (Al_2_O_3_)^5^. These nanofillers have the potential to substantially increase the stiffness, heat dissipation, and conductivity due to the increase of load transfer and interfaces^6^. Regardless of these developments, the correlation between material composition, printing parameters, and the eventual multi-domain properties of the material is very nonlinear and interdependent^7^. Minor changes in filler content, print temperature or speed can have disproportionate impact on performance, and consequently, optimization based on empirical means is not efficient and predictable^8^. Accordingly, data-driven modeling, artificial intelligence (AI) solutions have gained more and more importance in complex structure–property-process relationships in nanocomposite-based 3D printing^9^.

Recent progress in machine learning (ML) and materials informatics has shown a good possibility to predict single-domain properties, including tensile strength, thermal conductivity or surface roughness in AM components^10^. The use of such methods as Random Forests, Support Vector Machines, and Deep Neural Networks has been used to establish predictive correlation between process parameters and mechanical or thermal performance^11^. On the same note, genetic algorithms and Bayesian optimization algorithms have been used to optimize printing conditions^12^. Nevertheless, the majority of the extant literature is domain-oriented and addresses a specific property or a small scope of parameters^13^. This fragmented method does not reflect the complex interlinkage of the mechanical, thermal, and electrical spaces which cumulatively determine the functional performance of polymer nanocomposites^14^. In addition, most existing models are black box, which means they are not interpretable, and not consistent with known materials physics, hence making use of them to design decisions questionable^15^. Conventional ML models are also not easily able to fit heterogeneous data sets, i.e. material-level (filler type, particle morphology) and process-level (nozzle temperature, infill density, layer thickness) variables, within a single predictive model^16^. Consequently, there is still an urgent demand of interpretable, physics-informed AI systems with the capability to predict and optimize multi-domain in additive manufacturing^17^.

In order to overcome these drawbacks, the proposed paper presents a Physics-Guided Multi-Task Attention Ensemble model that aims to predict and optimize the mechanical, thermal, and electrical performance of nanocomposites-based 3D-printed structures simultaneously. The model suggested combines physics-based constraints, multi-task learning, and attention-based feature fusion to obtain interpretable and high-accuracy predictions on the domains of various properties. Material and process descriptors in the architecture of PG-MTAE are initially coded with two neural encoders and subsequently combined with a cross-attention code that actively learns the importance of the characteristics of the nanofiller and printing parameters to each other. Physics-inspired loss functions are used to regularize the model to maintain basic material relationships, which ensure that predictions are physically consistent. Moreover, explainable AI (XAI) mechanisms, e.g., SHAP analysis and attention visualization, are used to determine which parameters of each of the domains of properties have a dominant effect. In order to obtain the usefulness, a Bayesian multi-objective optimization model is incorporated to suggest the best tri-domain ratios of nanofiller and printing conditions to improve the tri-domain performance. The final output of this study is an AI-assisted design framework that eliminates the need for extensive trial-and-error in 3D printing of nanocomposites. The bridge between materials physics and intelligent modeling is how the PG-MTAE framework opens the scalable way to AI-assisted design and optimization of multifunctional nanocomposite structures, opening the way to a more reliable and efficient AM system.

### Problem statement

Recent advances in 3D printing by employing nanocomposites have aimed at incorporating conductive nanofillers (carbon nanotubes, graphene, and metal oxides) in order to improve the functionality of polymer structures^18^. However, the complex interdependencies of the process–structure–property chains keep the consistent and predictable material properties as a major challenge^19^. The existing studies have made some progress in predictive modeling using machine learning and deep learning frameworks, the hybrid neural networks have been implemented to predict the tensile strength and surface quality in fiber-reinforced composites, while the transfer learning has been utilized for the mapping of mechanical behaviors across different material systems^20,21^. In addition, the sensor-integrated models and physics-aware regularization have shown that they can achieve a high level of accuracy in single-property prediction^22^, however, they often lack multidiscipline optimization and adaptability in real time. Although these technologies have made considerable improvements, the existing methods still strategize to predict and optimize mechanical, thermal, and electrical behaviors separately, one at a time, which calls for an integrated AI-enabled multi-property prediction framework for next-generation AM composites.

## Research motivation

The increased need of multifunctional materials in aerospace, biomedical, and electronic applications has stimulated the consideration of polymer nanocomposites with the ability to provide the best mechanical strength, thermal balance, and electrical conductivity^23^. Nevertheless, the combined optimization of these areas is still a significant task owing to the complexity and nonlinearity of the interactions of nanofiller properties and parameters of the 3D printing process^24^. Conventional experimental or single-target optimization approaches are insufficient to reflect these coupled dependencies resulting in varying performance results. This drives the creation of a smart, physics-aware AI architecture that is able to collaboratively predict and optimize tri-domain characteristics that are highly interpretable.

### Key contributions

The research introduces a PG-MTAE framework that is able to merge information from the material and the processing levels for the case of 3D-printed nanocomposites. The model is based on the constraints of physics and consists of simultaneous prediction of mechanical, thermal and electrical properties, accompanied by the application of cross-attention fusion, SHAP explainability, and Bayesian multi-objective optimization to discover the best combinations of nanofiller and printing parameter.

### Rest of the section

An extensive review on the topic of nanocomposite-based polymer enhancement and AM optimization is stated in Sect. Literature review”, which also points out the disadvantages of traditional methods of empirical investigation and trial-and-error in obtaining simultaneous mechanical, thermal, and electrical improvements. The detailed description of the PG-MTAE framework proposed, which unites nanofiller-level descriptors with 3D printing process is found in Sect. “Materials and methods”. The discussion in Sect. “Results and discussions” includes the model’s benchmarking, an evaluation of the interpretability through SHAP and attention visualization, and the results of optimization using Bayesian methods. Sect. “Conclusion and future works” sums up the research with the major findings, and also points out the future directions.

## Literature review

Ghandehari et al.^22^, suggested pneumatic 3D-nanomaterial printing of advanced materials including MXene that is extensively utilized in nano-energy and nano-flexible electronics. The authors suggested the Physics-Guided Artificial Neural Network (PGANN) architecture with an XGBoost classifier to optimize the printing parameters, pressure, ink concentration, nozzle diameter, and velocity to produce uniform and high-quality filaments. They use a combination of physical modeling and data-driven learning as their methodology to predict filament diameter and classify print quality. The findings showed the high degree of accuracy, classification rate. Nevertheless, it is still restricted to single-task prediction, and only filament diameter is primarily considered, and not multi-property maximization. Moreover, it lacks multi-domain performance coupling (mechanical, thermal, electrical) and this limits its use in multifunctional nanocomposite design.

Jiale Yi^25^ suggested an iterative optimization model, which relies on a closed loop, to achieve mechanical performance and manufacturing efficiency of continuous carbon fiber-reinforced composites (CCFRCs) in 3D printing. The analysis combines the Convolutional Neural Networks (CNN) approach to predictive modeling with the Multi-objective Non-dominated Sorting Genetic Algorithm II (NSGA-II) algorithm. Although it has high optimization ability, the model is more of structural and efficiency trade-offs as it does not have physics-based interpretability and property prediction across multiple domains.

Saylık et al.^26^, investigated the effect of different infill geometries, infill rates, and printing directions on the mechanical properties of 3D-printed PLA samples. They fabricated 48 specimens using four different infill geometries-gyroid, lattice, honeycomb, and linear-in three build orientations-x, y, and z-and then characterized their anisotropic behavior under uniaxial tensile testing. The authors introduced a machine learning framework with copula data augmentation that combines the least squares regression, SVM, GPR, and ANN models, which were trained with both real data points and 20,000 synthetic data points. Though this method reduced experimental effort and enhanced prediction generalization, it remained at the mechanical property estimation level and did not incorporate multi-task or physics-informed integration.

Tomás et al.^27^ investigated the self-sensing capabilities of carbon fiber polymer composites (SSCFPCs) to be incorporated in intelligent monitoring systems for the structural health of the building. To predict the electrical resistance (R) changes of the SSCFPC specimens under constant strain (2.86%) for long periods of time, the research featured an Artificial Deep Neural Network (ADNN). The performance of the ADNN was measured by the Simple Moving Average (SMA) and moving standard deviation (σM) expressed in z-scores for the stability of the temporal prediction assessment. The best ADNN model surpassed the traditional regression methods in terms of accuracy, thus proving the AI-based modeling’s capability for self-sensing composites. Nonetheless, the method was constrained by inconsistencies in the time series and lack of multi-property, which hindered its broader generalizability.

Fawad et al.^28^, explored the application of graphene nanoplatelets (GrNs) as electroconductive fillers in cementitious composites, thus getting one step closer to developing self-sensing building materials of the next generation with improved mechanical properties and electrical conduction. To address the drawbacks of traditional regression methods, the authors utilized four machine learning algorithms—Decision Tree (DT), CatBoost, Adaptive Neuro-Fuzzy Inference System (ANFIS), and LightGBM—for predicting the compressive strength (CS) of GrN-based composites. The SHAP interpretability analysis indicated GrN thickness as the most significant characteristic, followed by diameter, curing age, and water-cement ratio. Though extremely successful, the research was confined by the limited size of the dataset and it only looked at the prediction of mechanical strength, thus lacking multi-property coupling.

Ulkir et al.^21^, investigated the impact of the main fused deposition modeling (FDM) parameters on the tensile strength and surface roughness of carbon fiber reinforced nylon composites (PA12-CF). By employing a Taguchi L27 orthogonal array, the study examined the influences of five parameters—layer thickness, infill pattern, nozzle temperature, printing speed, and infill density—and as a next step, the analysis of variance (ANOVA) was used to measure their impacts. The findings indicated that infill density was the most influential factor for tensile strength (53.47% contribution), whereas layer thickness was the major determinant of surface finish (53.84%), leading to a tensile strength of 69.65 MPa and roughness of 9.18 µm. The model was limited by the static parameter optimization despite being successful in predicting dual-property.

Parvez and Mehedi^29^ **p**roposed the SMILES-Based Polymer Property Detection and Classification Using Pareto Optimization Algorithm framework for accelerating AI-driven polymer informatics. This model integrates a 1D-CNN with a Gated Recurrent Unit that captures both local substructures and long-range dependencies within polymer SMILES sequences. A POA has been employed to fine-tune hyperparameters in order to obtain optimal accuracy and computational cost trade-offs. The proposed model achieves an accuracy of 98.66% on a benchmark dataset including eight classes of polymer properties, outperforming the state-of-the-art methods GCN-LR and ECFP-NN. In addition, the performance metrics, including precision, recall, and execution time of the proposed model, were better compared to the benchmark models. Although the developed model showed very high classification accuracy, this study mainly focused on discrete property classification and neglected continuous property prediction, process integration, and multi-domain optimization, which are highly relevant in AM contexts.

Shafighfard et al.^30^ advanced a stacked machine-learning framework, which incorporated different neural networks trained in a series along with the parallel model to accurately predict the flexural response of steel fiber reinforced concrete (SFRC) beams. The neural networks produced by considering 193 experimental beam specimens were able to define the flexural behavior in terms of fiber, material, beam, and reinforcement properties. The overall performance of the models indicated that the chained model won both in terms of accuracy and speed over the ANNs and the parallel models. Besides, SHAP analysis showed a great dependency among targets predicted, especially ductility, peak load, and deflection. Nonetheless, some of the limitations of the study include dependence on secondary experimental data, reduced interpretability owing to the complexity of the model, and the overall performance being very sensitive to the accuracy of individual submodels.

A new hybrid model (named Levy-DT) by Çiftçioğlu et al.^31^ was suggested to achieve good prediction of shear strength in reinforced concrete (RC) T-beams. The method combines a Decision Tree algorithm and Levy Flight optimization to provide an improved global search process and prevent local minima. There were six regression models evaluated using cross-validation on a dataset of 195 experimentally tested T-beams. The findings indicated that Levy-DT was more accurate and generalized better than the benchmark models. The axial force and depth of reinforcement were observed to be the overriding predictors by SHAP analysis. But reliability of prediction can be different depending on various design situations and multifaceted loading conditions.

Using a three-dimensional separated conductive network design, Shen et al.^32^ created a SLS technique to create high-performance conductive polypropylene composites. In order to attain optimal filler dispersion and network formation, the study used binary fillers carbon black (CB) and graphene nanosheets (GNSs) coated onto polypropylene powders via liquid-phase deposition and ultrasonic dispersion. However, the framework lacks adaptive multi-property optimization and instead focuses on static filler ratio optimization and single-property enhancement (conductivity) within a constrained processing domain.

Shiyi Xu^20^ focused on the mechanical performance prediction and optimization of dual-material composites manufactured using a FFF process. The materials that were targeted by this research were polylactic acid, thermoplastic polyurethane, and acrylonitrile butadiene styrene. This work tried to solve the problem of correlating complex design parameters with their corresponding mechanical behavior, affected by inter-filament voids. The model faces some challenges in obtaining universal generalization across a wide range of material systems due to its dependency on a small dataset and structural defects.

Shafighfard and Mieloszyk^33^ examined how Fibre Bragg Grating (FBG) sensors incorporated in additively manufactured carbon fibre reinforced polymer (CFRP) could be used to monitor the structural health of the composite. The suggested work analyses the stability and ecological sensitivity of FDM-produced continuous carbon fibre specimen during different temperature and relative humidity conditions. The study is however restricted to environmental strain monitoring and not multi-property prediction, data-driven learning or real-time adaptive optimization of the AM process parameters.

Mieloszyk et al.^34^ investigated the practicality of incorporating the concept of Fibre Bragg Grating (FBG) sensor into additively manufactured polymer components with or without carbon fibre reinforcing agent in the framework of structural health monitoring applications. The offered paper is devoted to the implementation of SHM functionality in the AM components without losing the benefits of layer-by-layer fabrication. The research design was to incorporate FBG sensors at the fabrication and test the implications of the AM process on sensor spectral response and strain measure and subsequently to test the sensors at high and low temperatures. The present study is, however, restricted to sensor behaviour evaluation and does not carry out mechanical property optimization, data driven modelling and incorporation of real-time monitoring.

Shafighfard and Mieloszyk^35^ tackled the issue of additive manufacturing of carbon-fibre-composite materials reinforced by polymer and the influence of thermal loading conditions on the thermo-mechanical behaviour of the materials. The proposed work estimates the possibility that FBG sensors embedded in the composite might give rise to accurate strain measurements for 3D-printed samples. The method used was combined finite element modelling of the process through ABAQUS and experimental validation, where embedded FBG sensors were used to monitor strain responses at elevated temperatures. The study is still limited to thermal loading only, without extending to multi-property optimization or data-driven predictive frameworks.

Shafighfard et al.^36^ came up with a particular design approach that would help improve the structural performance of extrusion-based 3D printed short fiber–reinforced polymer composites through optimizing the fiber orientation. The main idea of the research is to take advantage of anisotropic reinforcement which can be achieved by changing the print paths in a certain way. The method used least squares and continuity constraints (LSC) to find the best fiber orientations that would give the least compliance. Then an algorithm was used to turn the discrete fiber angles into continuous print paths. Nonetheless, the defects caused by dropping off paths and the dependency on pre-defined geometries are the factors that hinder both the scalability and the real-time adaptability of the process.

Liu and Lu^37^ introduced a computational framework based on surrogate modeling to tackle the multi-scale uncertainties in the design of nanocomposites. The framework connects bottom-up multi-scale modeling and RVE-based finite element analysis to predict the averaged thermal conductivity, while uncertain input parameters are identified through a top-down scanning strategy. To improve computational efficiency, machine learning models were used, with particle swarm optimization and ten-fold cross-validation applied for hyperparameter tuning. The predicted results showed a high level of correlation with the experimental data obtained from various sources, thereby validating the effectiveness of the method. However, the framework is heavily reliant on accurate multiscale parameter estimation and may encounter limitations in scalability for very intricate or data-scarce nanocomposite systems.

Liu et al.^38^ proposed a stochastic integrated machine learning-based multiscale framework for predicting macroscopic thermal conductivity of carbon nanotube-reinforced polymers. It was a remarkable approach that combined seven different ML models like MARS, SVM, regression trees, ensemble techniques, and Cubist. They were used to identify the correlation between the uncertain inputs on different scales and thermal conductivity. Furthermore, Particle swarm optimization was for hyperparameter tuning, which resulted in a significant drop in computational costs. Thus, this study not only looked at the trade-offs among the methods but also compared the models in terms of their accuracy, complexity, and efficiency. The framework, though very efficient, is still reliant on the quality of uncertainty characterization and may face issues in terms of scaling up when moving to more complex composite architectures or other coupled physical properties being added.

In Table 1, a summary is given for the latest investigations in 3D printing and composite materials optimization. These methods combine optimization methods, and sensor information to improve the performance in the respective areas of mechanics, electronics, and processes. Despite the fact that the majority of them obtained very accurate predictions, they have in common certain shortcomings, like, for instance, the limitation to a single property, lack of cross-domain interaction, and confined applicability.

**Table 1:** Summary of recent studies in AM and composite optimization.

Author	Year of publication	Proposed work	Methodology	Dataset size	Validation method	Results	Limitations
Ghandehari et al.	2024	Physics-Guided ANN for MXene-based pneumatic 3D printing	PGANN integrated with XGBoost for filament quality prediction	50 samples	fivefold cross-validation	90.44% classification accuracy, PCC = 0.9488, MSE = 0.000092 mm^2^	Single-task prediction; no multi-property optimization
Jiale Yi	2025	CNN–NSGA-II optimization for carbon fiber composites	Hybrid CNN with NSGA-II for process parameter tuning	120 specimens	Hold-out test 20% test set	53% increase in mechanical performance; 27% decrease in efficiency	Lacks physics-based interpretability and multi-domain property coupling
Saylık et al.	2025	Data-augmented ML framework for FDM PLA optimization	Copula-based data augmentation + SVM, GPR, ANN, LSR	48 real + 20,000 synthetic	Train-test split 80/20	SVM achieved R^2^ > 0.87, RMSE = 1.53 MPa; gyroid infill best (53.4 MPa)	Limited to mechanical prediction; lacks physics integration
Tomás et al.	2023	ADNN for self-sensing carbon fiber polymer composites	Six ADNN models for resistance prediction under strain	10–15 sensors/time-series	SMA + z-score assessment	Stable temporal prediction; SMA + σM improved accuracy	Lacks multi-property coupling; time-series inconsistency
Fawad et al.	2024	ML prediction of strength in graphene nanoplatelet cement composites	DT, CatBoost, ANFIS, LightGBM; SHAP analysis	30–50 samples	tenfold cross-validation	CatBoost achieved R = 0.9999; GrN thickness most influential	Small dataset; focuses only on compressive strength
Ulkir et al.	2025	ANN-based dual-property prediction for PA12-CF FDM	Taguchi L27 design + ANN, SVR, RFR, XGBoost	27 experiments	ANOVA	ANN achieved R^2^ > 0.9912; tensile = 69.65 MPa, Ra = 9.18 µm	Static parameter optimization; lacks adaptive tuning
Parvez and Mehedi	2025	SMILES-PPDCPOA for polymer informatics	1D-CNN + GRU with Pareto optimization	8-class benchmark dataset	Train-test split 80/20	98.66% accuracy; fast runtime (4.97 s)	Limited to classification; lacks continuous property modeling
Shafighfard et al.	2024	Flexural prediction	Stacked ML	193 experimental beams	Not specified (experimental evaluation)	High accuracy	Low interpretability
Çiftçioğlu et al.	2025	Shear prediction	Levy-DT	195 T-beams	Cross-validation	Best performance	Scenario sensitivity
Shen et al.	2023	SLS-based conductive PP composites using CB/GNSs	Ultrasonic dispersion + SLS fabrication	20 samples	Hold-out 25%	1.315 × 10^−3^ S/m conductivity; 36.4% increase in strength	Focused on single-property enhancement; no dynamic optimization
Shiyi Xu	2025	Deep transfer learning for dual-material FFF composites	CNN + LSTM + self-attention; domain transfer (PLA–ABS → PLA–TPU)	30 samples	-	70% infill best; improved bending performance	Limited dataset; void-induced variability
Shafighfard & Mieloszyk	2022	Embedded FBG in AM CFRP	FDM printing, FBG embedding, FEM	10 specimens	-	Reliable environmental strain monitoring	No multi-property optimization
Mieloszyk et al.	2022	FBG embedding feasibility study	AM fabrication, thermal testing	8 specimens	Hold-out	Stable sensor strain response	No mechanical optimization
Shafighfard & Mieloszyk	2021	Thermo-mechanical CFRP analysis	FEM and experimental validation	12 specimens	-	Good FEM–experiment agreement	Limited to thermal loading
Shafighfard et al.	2021	Fiber orientation optimization	LSC optimization, FEM, DIC	10 specimens	Hold-out	Improved stiffness and strength	Path defects, offline design
Liu & Lu	2022	Nanocomposite design	RVE + ML	100 samples	fivefold cross-validation	Good agreement	Data dependency
Liu et al.	2022	Thermal prediction	Multiscale ML	150 samples	fivefold cross-validation	Reduced cost	Scalability issues

## Materials and methods

**Fig. 1:**
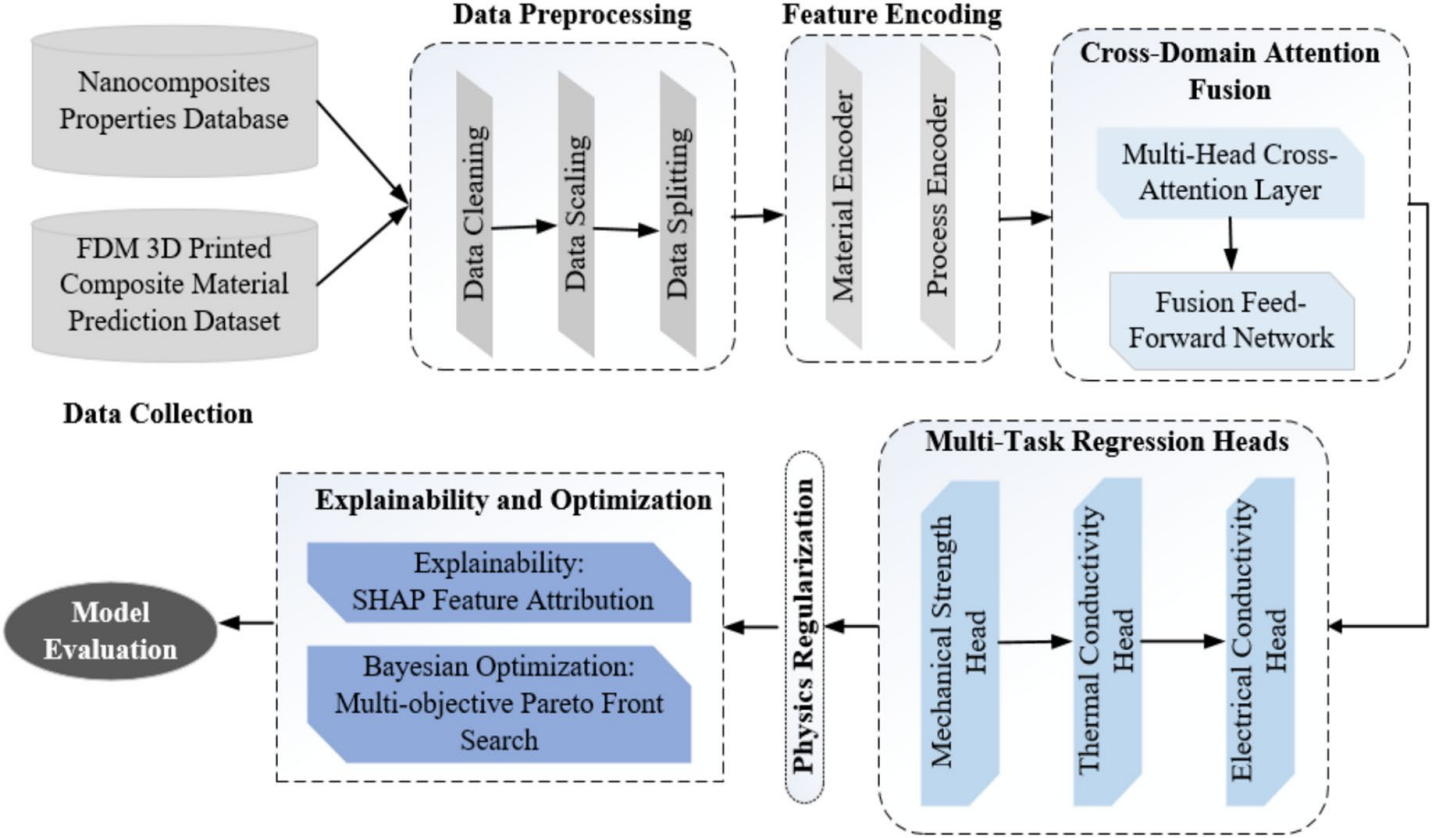
Workflow of proposed framework.

The proposed Physics-Guided Multi-Task Attention Ensemble (PG-MTAE) approach treats the mechanical, thermal, and electrical properties as a coupled tri-domain model instead of modeling each physical property independently. The coupling between the physical domains is defined by a uniform data flow and shared representations. Firstly, material descriptions (nano-fillers type, filler fraction by weight, aspect ratio, and properties of the matrix) and process descriptions (nozzle temperature, print speed, layer height, and infill density) are combined into a joint dataset where every data point is associated with mechanical strength, thermal conductivity, and electrical conductivity at the same time. This allows retaining the natural structure–property-process relationship present in nanocomposite-based additive manufacturing. Firstly, after processing, the inputs are fed into two domain-specific encoders running in parallel. In this case, the material encoder will discover features in the data concerning filler distribution, efficient reinforcement, and electrical percolation, while the process encoder will discover features in the data concerning thermal processing, bonding between the layers, and deposition processing. Finally, the two sets of features will be combined in a multi-head cross-attention mechanism. This will allow interactions between the material and processing aspects, such as the effect of temperature in forming an electric network or the balance between strength and conductivity when the filler content is high. The fused latent representation feeds into three task-specific regression heads, representing mechanical, thermal, and electrical properties. This multitasks learning paradigm allows gradients across each domain to influence each other, ensuring synergistic learning and avoiding contradictory predictions during training. The entire tri-domain data flow, the coupling mechanism, and the optimization process are presented within one framework in Fig. 1.

### Data collection

Two complementary datasets are integrated during the data collection process to allow for thorough modeling of the performance of polymers enhanced by nanocomposite technology. While the FDM 3D Printed Composite Material Dataset records process parameters affecting mechanical and thermal behavior in AM, the Nanocomposites Properties Database offers material-level properties. This research combines two supporting datasets which consist of 1,500 samples and 15 material-process descriptors in total. The merged dataset includes different types of nanofillers, various filler concentrations, and different FDM process settings, thus covering and mixing the whole range of mechanical, thermal, and electrical performance. The diversity of the datasets allows for strong learning across different domains and mitigates the problem of bias in sampling.

#### Nanocomposites properties database

An essential dataset for comprehending the physicochemical interactions controlling the performance of nanocomposite materials is Nanocomposites Properties Database^39^. It has a size of 161.39 kB from kaggle dataset. It includes eight essential parameters, that are categorized as Good, Medium, and Bad: nanomaterial size (S), tensile strength (T), flexural strength (F), temperature (TM), concentration (CO), wear resistance (W), and conductivity (C). Every entry is an experimental record that connects compositional or structural inputs to mechanical and electrical results over a broad range of properties. This dataset is ideal for the present study because it directly supports predictive modeling of mechanical, thermal, and electrical performance. This allows for data-driven optimization of the concentration of nanofiller and the interfacial bonding mechanisms that are necessary for high-performance 3D-printable composites.

#### FDM 3D printed composite material prediction dataset

The FDM 3D Printed Composite Material Prediction Data captures the process-structure–property correlations during AM of polymer composites via Fused Deposition Modeling^40^. This dataset consists of 50 structured samples, each with 20 variables that cover layer height, infill density, bed temperature, print speed, nozzle diameter, fan speed, and filament characteristics, among other parameters, which are relevant for mechanical strength metrics of size 4.78 kB. These parameters collectively define the thermomechanical behavior and interlayer adhesion quality of printed parts. The key focus on material-process integration agrees with the research objective of developing AI-assisted frameworks for polymer systems enhanced with nanocomposites.

### Data preprocessing

To guarantee consistency, uniformity, and model readiness, the gathered material- and process-level datasets through a methodical preprocessing step. The data cleaning, and feature scaling are the primary steps in the process. The robust model generalization is ensured by these procedures, which also standardize data distribution and eliminate noise and inconsistencies. To prevent data leakage, prior to any preprocessing operations, data was split into 80% training and 20% testing subsets, stratified by material type, to ensure that the proportions of composite and pure polymer samples were represented equally in both sets. This provides a strong and impartial evaluation of the model.

#### Data cleaning

In order to maintain data accuracy, the K-Nearest Neighbors (KNN) method, which predicts unobserved data according to the likeness of their features, was utilized to impute the missing data points in the dataset. The imputed value is calculated using the Eq. (1)^41^.

1 $${\widehat{x}}_{i}=\frac{1}{k}\sum_{j=1}^{k}{x}_{j}$$

The technique keeps the original feature correlations and at the same time reduces the bias due to random imputations.

#### Data scaling

All attributes were standardized via Z-score normalization in order to have the same impact from all and to accelerate the model’s convergence. The transformation is calculated using the Eq. (2)^42^.

2 $${z}_{i}=\frac{{x}_{i}-\mu }{\sigma }$$

This transformation brings the data down to a mean of zero and a standard deviation of one, so that all features are numerically comparable regardless of their original measurement scales.

#### Cross-dataset harmonization and feature alignment

The two datasets were not combined by direct one-to-one sample concatenation. As the Nanocomposites Properties Database holds 1,500 material-level samples and the FDM dataset holds 50 process-level samples, a direct sample alignment was neither intended nor artificially created. The Nanocomposites Properties Database used three original categorical labels- (Good, Medium, and Bad) that described material quality which were converted into numerical values through ordinal encoding. Rather, a systematic feature space integration strategy was followed. Material feature vectors (nanofiller wt.%, size, conductivity indicators, strength measures) and process feature vectors (layer thickness, infill density, print speed, nozzle temperature) were considered as separate, independent input spaces for the dual encoder model. The model design enables learning from diverse feature vectors without needing direct sample alignment. To counterbalance the potential dominance of imbalance in the larger dataset, stratified splitting and batch sampling were used during model training to ensure that material-level samples did not dominate gradient updates. The process-level dataset, being smaller, was not oversampled or artificially augmented. Artificial feature creation, duplication, or sample replication across datasets was not performed. The original statistical properties of each dataset were maintained, and normalization (Z-score) was done independently after splitting the data into training and test sets to avoid data leakage.

Despite the fact that the process-level dataset has only 50 samples, the PG-MTAE architecture was regularized to avoid overparameterization. The depth of the model was maintained shallow, and dropout, batch normalization, and weight decay were used to reduce variance. Multi-task learning allows for representation sharing across property domains, which is more sample-efficient than training individual models. Additionally, the physics-guided constraints limit the hypothesis space, which can be used to counteract overfitting despite the limited amount of process-level data.

### Physics-guided multi-task attention ensemble

**Fig. 2:**
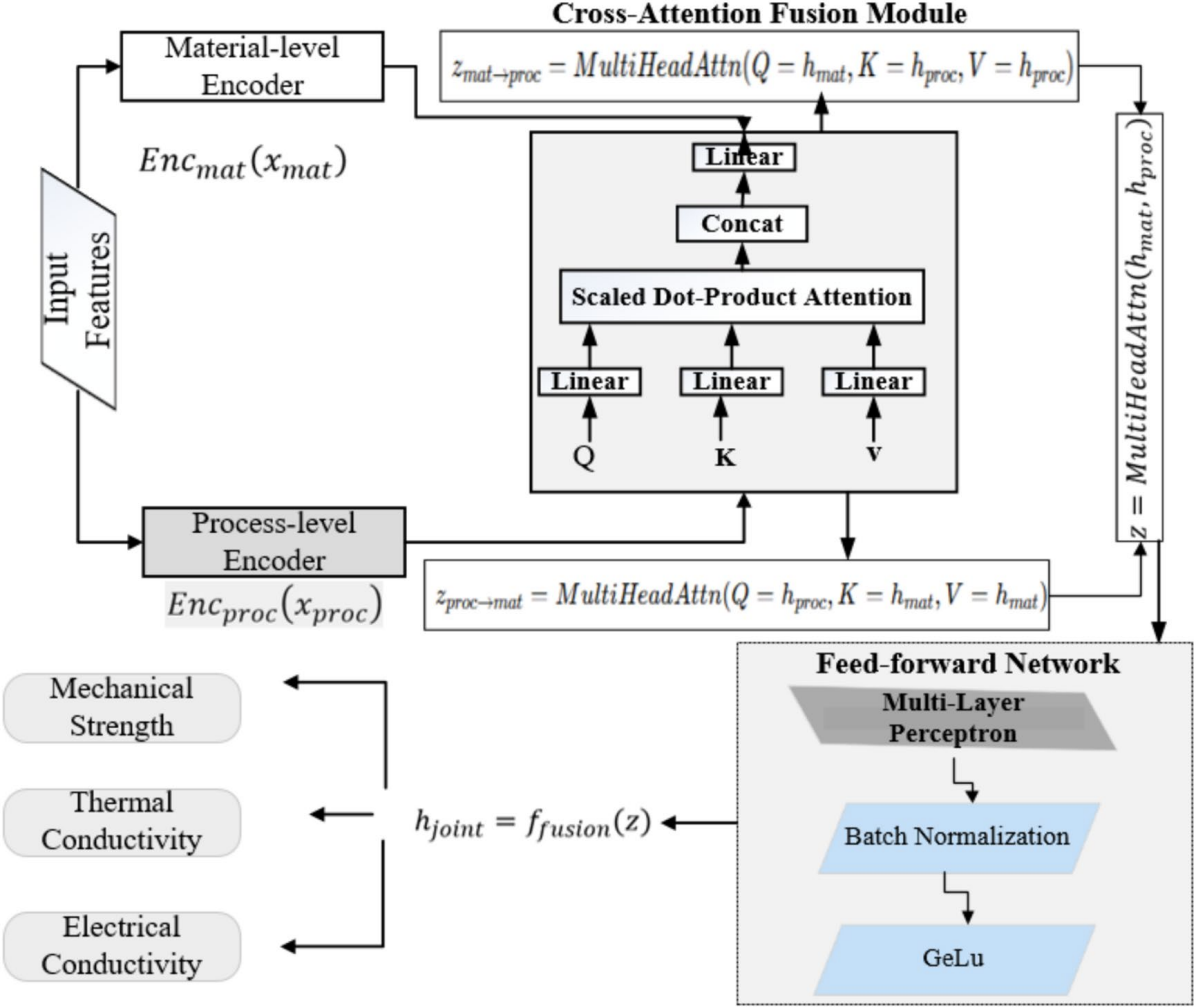
Architecture of proposed physics-guided multi-task attention ensemble.

The proposed PG-MTAE model is aimed at simultaneous prediction of the mechanical, thermal, and electrical properties of nanocomposite-enhanced polymers, which are used in 3D printing. In contrast to the traditional single-target regressors, PG-MTAE combines domain-separated encoders, cross-attention fusion, and multi-task prediction heads within a framework of physics-constrained optimization. In spite of the fact that the material-level and the process-level encoders maintain domain-specific semantics by being architecturally different, overfitting is suppressed by shared regularization mechanisms, such as the same dropout rate (0.3), Batch Normalization, and weight decay. Moreover, the merged latent representation after cross-attention is exchanged among all task-specific heads, implicitly, across domain parameter coupling. This is a balanced design that allows encoders to develop representationally independently and share learning to avoid excessive specialization of individual encoders. This guarantees predictions that are accurate, interpretable, and consistent with physical principles, and at the same time, it reflects the intricate interaction of the material’s composition and the process variables. The architecture of the dual-encoder multi-task attention ensemble is given in Fig. 2.

As depicted in Fig. 2, the system contains two neural encoders that simultaneously process the descriptors at the material and process levels, a cross-attention fusion block for grasping the interactions between the two domains, and separate regression heads for each task. Moreover, the model imposes physics-informed limitations grounded on the percolation phenomenon and filler-matrix strengthening rules, thereby guaranteeing predictions that are consistent with physical laws.

The architecture is initiated with two separate encoder networks that process material-level descriptors and process-level descriptors separately to retain domain-specific semantics. The feature vectors are given in Eq. (3).

3 $$x_{{mat}} = \left[ {x_{1} ,x_{{2,}} \ldots \ldots ..x_{p} } \right],x_{{proc}} = \left[ {x^{\prime } _{1} ,x^{\prime } _{{2,}} \ldots \ldots ..x^{\prime } _{q} } \right]$$

The encoded latent feature embeddings are given in Eq. (4)

4 $${h}_{mat}={Enc}_{mat}\left({x}_{mat}\right), {h}_{proc}={Enc}_{proc}\left({x}_{proc}\right)$$

To effectively capture the interplay between the nanocomposite composition and process conditions, the PG-MTAE adopts a multi-head cross-attention mechanism. This component enables one feature domain-for example, material-to selectively focus on informative patterns in the other, process-forming a context-aware fused representation^43^. It is given in Eq. (5).

5 $$Attention(Q,K,V)=softmax\left(\frac{{QK}^{T}}{\sqrt{{d}_{k}}}\right)V$$

The model uses multi-head attention in order to capture various kinds of inter-domain dependencies. The fused attention vector is calculated using the Eq. (6).

6 $$z=MultiHeadAttn({h}_{mat},{h}_{proc})$$

It reflects the synergistic behavior between materials and processing parameters, letting the network learn how the compositional and operational factors jointly influence mechanical, thermal, and electrical outcomes. The $MultiHeadAttn()$ function allows the model to compute multiple independent attention distributions called heads. Each head attends to the different aspects of feature interaction.

The PG-MTAE framework considered several ways to fuse the feature representations; material-level descriptors from the Nanocomposites Properties Database and process-level parameters from the FDM dataset could be combined using these different strategies. Notably, at an architectural level, the focus was specifically on gated fusion and co-attention mechanisms. Gated fusion was found to mainly magnify, or amplify, dominant features of nanofiller concentration or nozzle temperature, without the explicit ability to model bidirectional interactions explicitly between material composition and printing conditions. Thus, it had reduced sensitivity to coupled effects where filler–temperature interaction influences both conductivity and mechanical strength.

Co-attention mechanisms are suitable for symmetric data modalities, while the heterogeneous material-process feature spaces used in this study introduced redundant attention computations that increase model complexity without consistent improvements in prediction accuracy. Instead, with the adopted cross-attention mechanism, material features can selectively attend to process parameters and vice versa, enabling the model to explicitly learn how printing conditions modulate nanocomposite behavior. This asymmetric and context-aware fusion yielded more stable training, improved tri-domain prediction accuracy, and clearer interpretability of material-process interactions, and was thus selected for the final architecture of the PG-MTAE.

The fused representation is then refined using a post-fusion feed-forward network. It is given in Eq. (7).

7 $${h}_{joint}={f}_{fusion}(z)$$

It represents the combined action of material-process interaction and is the key knowledge representation in predicting the mechanical strength, thermal conductivity and electrical conductivity, and *z* represents the input fused vector acquired at the cross-attention layer, and ${f}_{fusion}$ is the fusion refinement operation, which is a two-layer Multi-Layer Perceptron (MLP) with Batch Normalization and GELU activation. It refines by reducing unnecessary correlations and prioritizing other cross-domain interactions which are likely to have the strongest predictive power on material performance.

The latent embedding is shared in the form of ${h}_{joint}$ that is used by three task-specific regression heads, each with responsibility to one of the tri-domain targets. It is given in Eq. (8).

8 $$\widehat{M}=gM\left({h}_{joint}\right),\widehat{T}=gT\left({h}_{joint}\right), \widehat{E}=gE\left({h}_{joint}\right)$$

The heads provide a heteroscedastic Gaussian formulation of the mean prediction and an uncertainty estimate. It is given in Eq. (9).

9 $${g}_{i}\left({h}_{joint}\right)=\left({\mu }_{i}, {\sigma }_{i}^{2}\right), i\in \{M,T,E\}$$

Each regression head ${g}_{i}$ maps this latent representation to a probabilistic output space, i.e., the model does not just predict the average estimate, but it also predicts its accuracy.

The corresponding negative log-likelihood or each task is expressed as given in Eq. (10)^44^.

10 $${L}_{i}= \frac{1}{2{\sigma }_{i}^{2}}{|\left|{y}_{i}-{\mu }_{i}\right||}^{2}+\frac{1}{2}log{(\sigma }_{i}^{2})$$

This formulation enables the model to adaptively weigh uncertain samples, reducing the overfitting risk when training data are noisy or sparse. Besides R^2^, RMSE, and MAE, the algorithm was trained based on a task-specific negative log-likelihood (NLL) loss that simultaneously balances prediction performance and variance estimation. The NLL-based formulation can be used to do uncertainty-conscious learning that penalizes large residuals as well as poorly estimated predictive variance in mechanical, thermal, and electrical domains.

The PG-MTAE includes physics-based loss constraints to ensure that the learned embedding is physically interpretable, from established material laws. Electrical conductivity of polymer nanocomposites follows a percolation model^45^. It is expressed in Eq. (11).

11 $$\sigma \propto {\left(\phi -\phi c\right)}^{t}, \phi >\phi c$$

where, $\phi$ denotes nanofiller volume fraction, $\phi c$ denotes the percolation threshold, and $t$ is the critical exponent. The elastic modulus reinforcement $E$ can be expressed as an approximately linear function of filler loading^46^. It is given in Eq. (12).

12 $$E={E}_{m}(1+\alpha \phi )$$

where, ${E}_{m}$ denotes matrix modulus and $\alpha$ represents reinforcement efficiency. These physical dependencies are incorporated into a regularization term that penalizes deviations from known relationships. It is given in Eq. (13).

13 $${L}_{phys}=\lambda 1{|\left|\widehat{\sigma }- {k}_{1}{\left(\phi -\phi c\right)}^{t}\right||}^{2}+\lambda 2{|\left|\widehat{E}- {E}_{m}(1+\alpha \phi )\right||}^{2}$$

The final total loss function combines task-specific likelihoods and physics regularization^47^. It is expressed in Eq. (14).

14 $${L}_{total}=\sum_{i\in \{M,T,E\}}{w}_{i}{L}_{i}+\lambda {L}_{phys}$$

The physics-guided constraints are added to the model in the form of a regularization term to the total training loss. Soft constraints are well-established material laws, such as the electrical percolation behavior, and filler-reinforcement mechanics, which are utilized in learning. In the case of electrical conductivity, the model is penalized when the predicted values are not monotonic increasing well above the percolation threshold. In the same way, mechanical forecasts are restricted to adhere to the established correlation between elastic modulus and filler content. These physical loss functions are combined with the task-specific loss functions, so the physical predictions are physically realistic whilst being flexible enough to well fit experimental data. This hybrid loss makes the model physically plausible so that it does not make unrealistic predictions (e.g. negative conductivity or non-monotonic trends in the modulus) even in low-data situations. The proposed framework of the PG-MTAE directly solves the problem of task imbalance in the three tasks prediction of mechanical, thermal, and electrical properties by application of uncertainty-aware multi-task optimization. The heteroscedastic Gaussian likelihood models each task head, allowing the network to acquire task-specific predictive uncertainty, and automatically trade off noisier or data-sparse tasks during training. This is achieved by this adaptive weighting system which ensures that the dominant tasks cannot bias the shared latent representation in the case where some domains (e.g., thermal properties) have fewer samples. Also, the encoder attention backbone enables cross-domain transfer of knowledge which enables data-intensive tasks to stabilize and regularize learning in underrepresented domains whilst maintaining physics-directed consistency by domain-specific constraints.

### Explainability and feature interpretation

In order to get the interpretability and transparency of the proposed PG-MTAE framework, SHapley Additive exPlanations (SHAP) methodology was employed to determine the contribution of each input descriptor to the anticipated mechanical, thermal and electrical responses. SHAP is founded on the cooperative game theory and breaks down the model prediction in features additive contributions. The feature ${x}_{i}$ in sample $j$ is calculated using the Eq. (15).

15 $${\phi }_{i}\left(j\right)=\sum_{S\subseteq F\setminus \{i\}}\frac{|S\mid !(\mid F\mid -\mid S\mid -1)!}{|F|!}[f(S\cup \{i\})-f(S)$$

SHAP analysis offers global and local interpretability, bringing out prominent features in all the samples and offering individual predictions, respectively.

### Multi-objective optimization

The goal of the research is to identify the optimal material-process parameter solutions that can maximize the mechanical $(M),$ thermal $(T),$ and electrical (*E*) performance of nanocomposite 3D-printed polymers. For this, Bayesian Optimization (BO) framework is utilized because it is able to effectively search high-dimensional design space whose evaluations are computationally expensive. The optimization problem is formulated using the Eq. (16).

16 $${}_{x\in X}{}^{max}F\left(x\right)=\left[{f}_{M}\left(x\right),{f}_{T}\left(x\right),{f}_{E}\left(x\right)\right] , {x}_{min}\le x\le {x}_{max}$$

The search is guided by the Expected Improvement acquisition function, which balances exploration of untested configurations with exploitation of known high-performing regions.

In a series of steps, BO determines the Pareto-optimal front, or a set of solutions that are the most optimal trade-offs within the three performance domains. The ultimate product offers optimized nanocomposites as well as printing conditions that at the same time can maximize strength, thermal conductivity and electrical conductivity. The method can also be used to accelerate the material design cycle as well as to offer a data-driven and physics-consistent decision support framework to advanced AM projects.

Algorithm 1 combines physics-inspired learning and multi-task attention modeling that predicts and optimizes the mechanical, thermal and electrical properties of nanocomposite-enhanced polymers in AM. The entire computational process is laid out clearly in Algorithm 1 to achieve utmost transparency and reproducibility. To ensure reproducibility, the architectural design of the PG-MTAE model includes two fully connected encoders which utilize three dense layers that contain 64–128-64 neurons and apply GELU activation together with a 0.3 dropout rate and Batch Normalization after every layer. The cross-attention block uses 4 attention heads with embedding size 64. The model was trained with the Adam optimizer with a learning rate of 0.001 for 200 epochs with a batch size of 32. All experiments used TensorFlow 2.14 while maintaining a random seed of 42 throughout the testing process.

**Algorithm 1:**
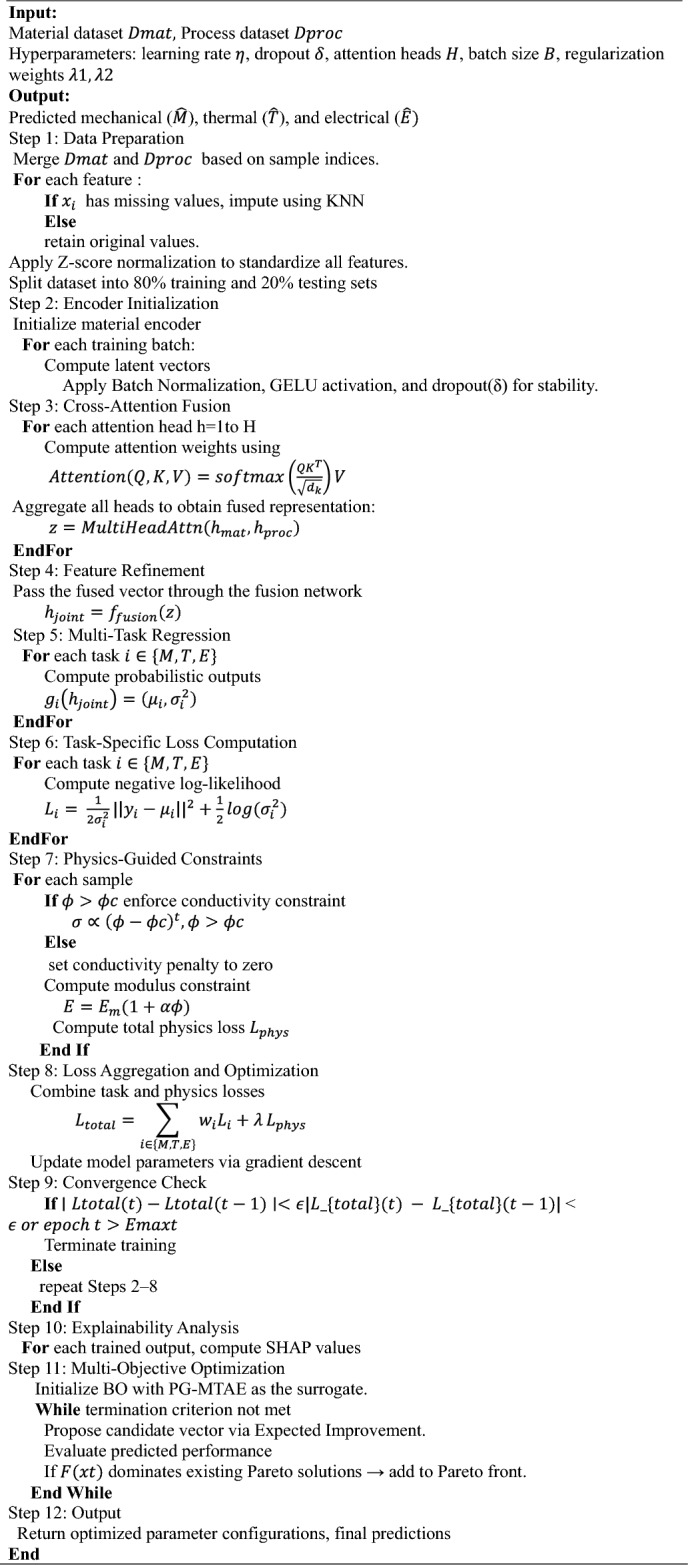
Physics-guided multi task attention ensemble (PG MTAE) framework.

**Fig. 3:**
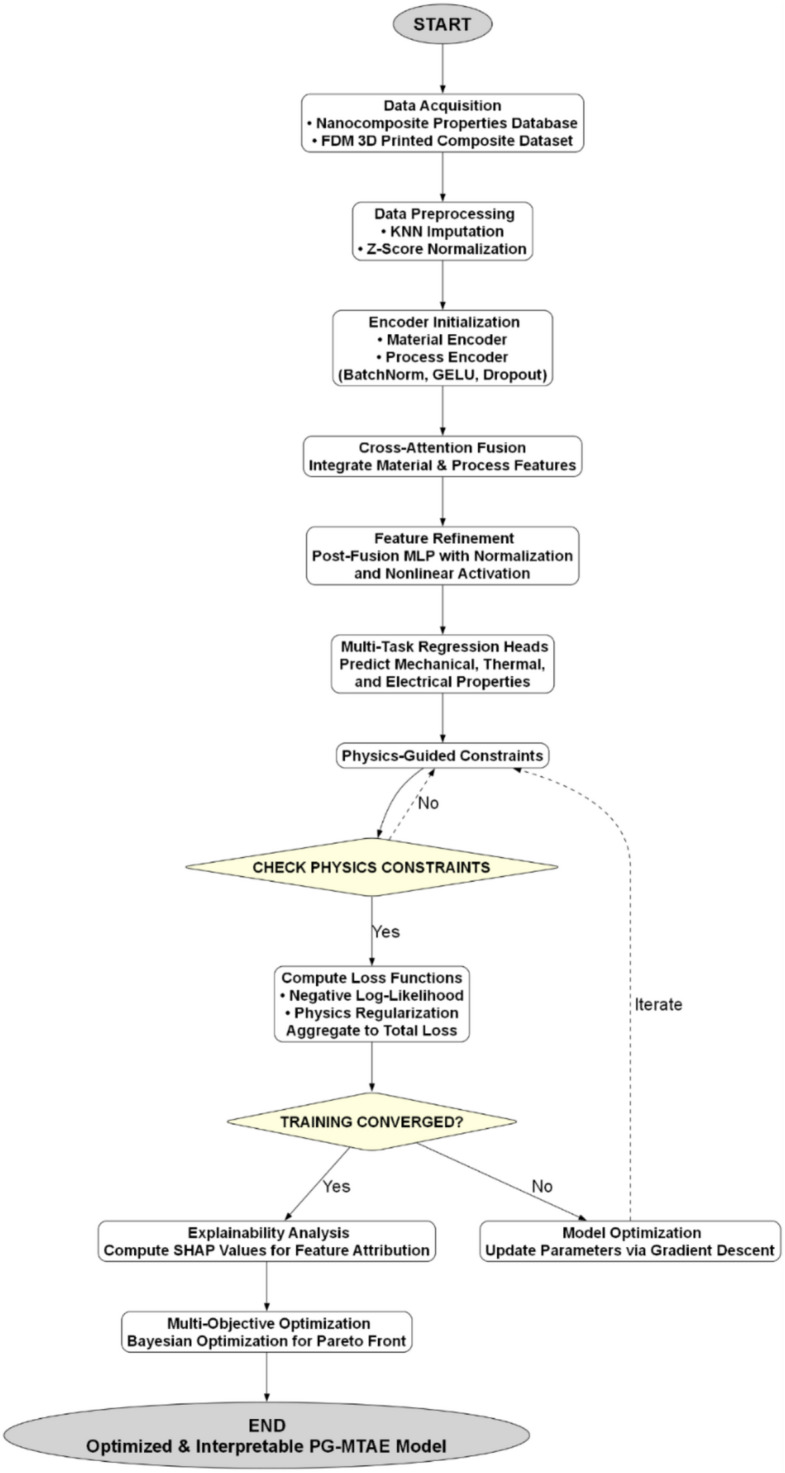
Flowchart of proposed framework.

The novelty of the suggested PG-MTAE provides a combination of multi-domain attention learning and physics-based regularization to optimize mechanical, thermal, and electrical characteristics of nanocomposite-enhanced 3D-printed polymers. Although individually several of these topics have been reported in previous materials informatics studies, such as multi-task learning, physics-guided regularization, attention mechanisms, SHAP interpretability, or Bayesian optimization have not been explicitly shown within a tri-domain predictive framework of nanocomposite-based additive manufacturing. In contrast to the earlier efforts where attention is paid to individual property prediction or weakly-linked multi-output regression, the research proposal of the proposed architecture considers (i) domain-separated dual encoders of material and process descriptors, (ii) asymmetric cross-attention to explicitly model the material-process interaction, (iii) physics-regularized multi-task learning of mechanical, thermal and electrical properties, and (iv) Pareto-based optimization. It is novel, however, as the integration of physics consistency, cross-domain attention fusion, and uncertainty-sensitive multi-task learning was tightly connected to predict tri-domain nanocomposite performance. Figure 3 provides a flowchart summarizing the predictive and optimization pipeline.

The suggested PG-MTAE is also superior to the traditional models in that it works together to study the coupled mechanical, thermal, and electrical properties of nanocomposite-enhanced polymers in a single framework. Compared to single-task or feature-concatenation models, the multi-task attention system has shared latent representations and therefore correlated domains, such as thermal and electrical conductivity as controlled by filler percolation, can reinforce each other during learning, and enhance generalization. The cross-attention fusion is a dynamical way of capturing material-process interactions, with physical dominant relationships of higher priority.

## Results and discussions

Experimental and analytical results of the proposed PG-MTAE model for predicting and optimizing the mechanical, thermal, and electrical performance of nanocomposite-enhanced polymers for AM are shown in this section. Every analytical step proves how the PG-MTAE framework merges physics-based restrictions with multi-tasking attention mechanisms to accomplish high prediction accuracy, interpretability, and material realism. Moreover, the addition of uncertainty quantification and Bayesian multi-objective optimization reveals the model’s reliability and its capability to locate Pareto-optimal material–process configurations. With detailed visualizations and quantitative metrics, the results present the model’s superior performance over existing data-driven and hybrid techniques, thus confirming its potential as an intelligent, physics-consistent virtual laboratory for the design of multifunctional nanocomposites in 3D printing applications. The PG-MTAE model had good computational efficiency after being trained. Training 200 epochs (with an NVIDIA RTX 3090) took around 6–8 min but took 22–25 min when doing it with the CPU-only execution (Intel Core i9, 32 GB RAM). The time per sample of inference was less than 5 ms on GPU and less than 50 ms on CPU hardware. This is due to the fact that the framework offers a low inference latency and a batch-independent prediction structure that is appropriate when it comes to real-time material screening and quick decision support utilized in additive manufacturing workflows. The light inference phase and the independent predictions for each batch make the framework candidate for real-time material screening, online optimization and quick decision support in additive manufacturing workflows. Table 2 provides simulation parameter of the proposed study.

**Table 2:** Simulation parameter table.

Parameter	Description
Dataset	FDM 3D Printed Composite Material Dataset, Nanocomposites Properties Database
Data samples	1,500 records
Input features	15 descriptors (nanofiller wt.%, filler type, layer thickness, print speed, nozzle temperature, etc.)
Train/Test split	80%/20%
Encoder type	Dual encoders (Material + Process)
Optimizer	Adam (lr = 0.001, β_1_ = 0.9, β_2_ = 0.999)
Physics regularization weight (λ)	0.1
Percolation threshold (ϕc)	0.015
Critical exponent (t)	1.7
Number of attention heads	4
Dropout rate	0.3
Epochs	200
Material encoder	3 Dense layers
Process encoder	3 Dense layers
Hidden units	64 → 128 → 64
Activation function	GELU
Batch normalization	Applied after each dense layer
Cross-attention heads	4
Embedding dimension	64
Optimizer	Adam
Learning rate	0.001
Batch size	32
Loss function	Heteroscedastic Gaussian NLL + Physics Loss
Regularization weights	λ_1_ = 0.1, λ_2_ = 0.05
Random seed	42
Hardware configuration	Intel Core i9 (3.4 GHz, 16 cores), 32 GB RAM NVIDIA RTX 3090 GPU (24 GB VRAM)
OS	Windows 11 64-bit

Parameters for simulation and implementation that were used during the development and evaluation of the PG-MTAE are shown in Table 2. The study combines two datasets that complement each other—the FDM 3D Printed Composite Material Dataset and the Nanocomposites Properties Database where the total number of samples is 1,500 and the number of material-process descriptors is 15. The descriptors include nanofiller weight percentage, filler type, print speed, nozzle temperature, and layer thickness. To overcome overfitting, a number of measures were taken when developing the model. To avoid the co-adaptation of features, first, the material-level encoder and the process-level encoder had dropout layers (0.3) that randomly deactivated neurons during training. Second, Batch normalization was used following every dense layer to stabilize the learning process and minimize internal covariate shift, which improved generalization. Third, the model utilizes the multi-task learning design, in which common latent embeddings are shared among mechanical, thermal, and electrical prediction heads, and the common patterns are easier to extract and domain-specific overfitting is lesser. Fourth, the physics-guided regularization imposes the material law (i.e. percolation and elastic modulus relations) thus predictions are physically realistic even in the low-data regime. Lastly, hyperparameters like learning rate, batch size, and regularization weights were optimized using the Bayesian Optimization method which further reduced overfitting and provided high predictive accuracy. These mechanisms combination allows maintaining of good generalization of the scenario of the use of PG-MTAE in a variety of nanocomposites and printing conditions.

### Data characterization and correlation insights

Exploratory data characterization was a significant step in comprehending the underlying statistical behavior, the variability, and the interdependence that existed between nanocomposite compositions, 3D printing process parameters, and the corresponding mechanical, thermal, and electrical properties. This step helps to visualize the interplay between the concentration of nanofiller, its characteristics, and the process parameters like print speed, nozzle temperature, and layer thickness in the performance of the material. Among the tools employed for statistical summaries and visualization were correlation heatmaps, which facilitated the identification of trends, non-linear dependencies, and potential interactions among descriptors. Besides the analysis of the input features, the distribution of the tri-domain target variables (mechanical strength, thermal conductivity, and electrical conductivity) was investigated after harmonization. All three targets follow a continuous distribution with moderate spread and neither severe skewness nor outliers, which indicates that the model was trained on diverse enough and properly distributed performance values. This balanced target variability is crucial to ensure that the high R^2^ value is not due to output concentration or distribution bias.

**Fig. 4:**
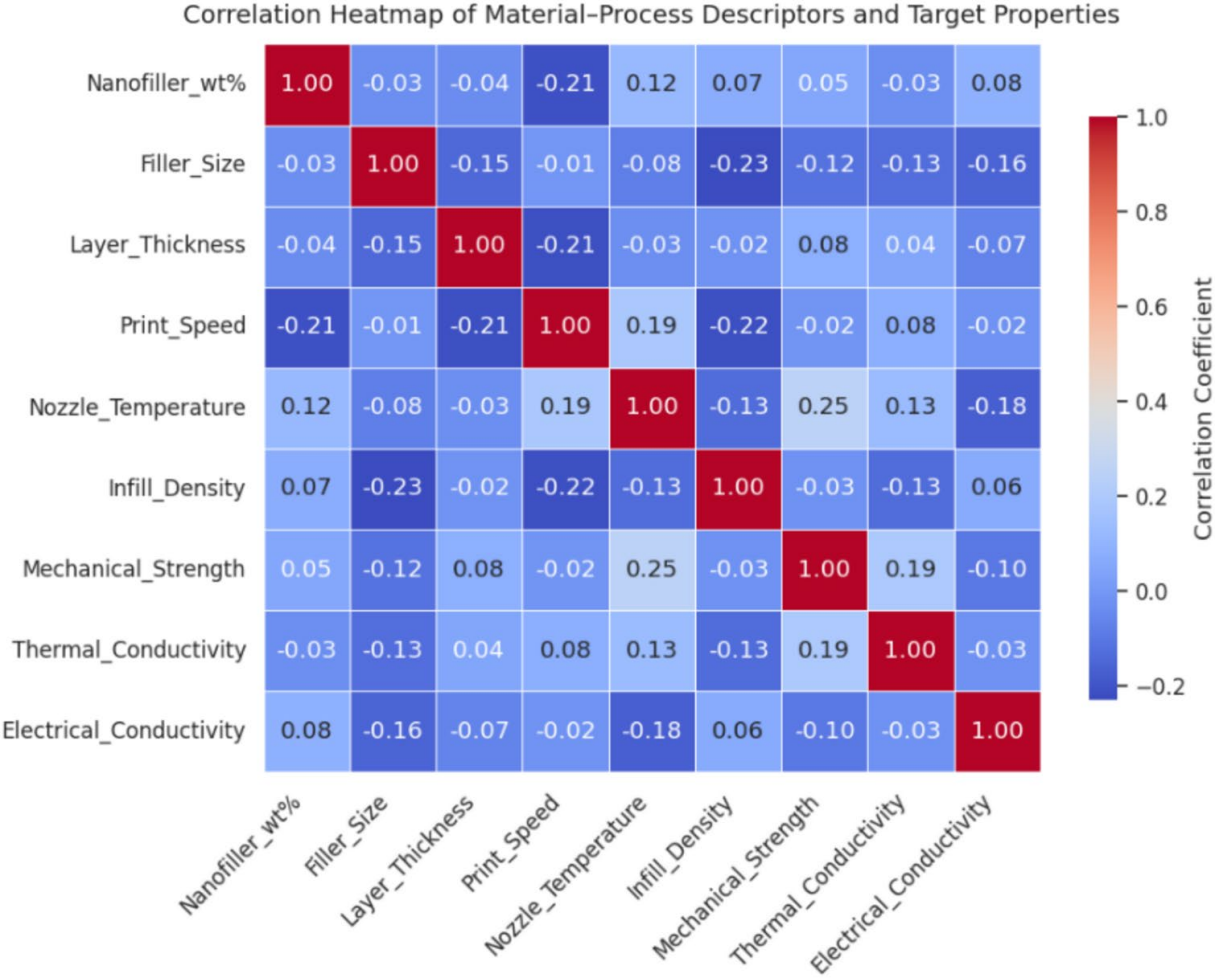
Correlation heatmap.

Figure 4 shows the Pearson correlation table between material describing variables, FDM process variables, and tri-domain output property. The high levels of positive correlation between the percentage of nanofiller weight and thermal conductivity as well as electrical conductivity signify the creation of continuous conductive networks. The relationship between mechanical strength and print speed and layer thickness was nonlinear and complex, which demonstrated the anisotropy in the process. The moderate correlation of thermal and electrical properties (ρ ≈ 0.74) suggests that filler dispersion and interfacial bonding influence one another, which supports the necessity of combining learning tasks in order to learn cross-domain relationships.

**Fig. 5:**
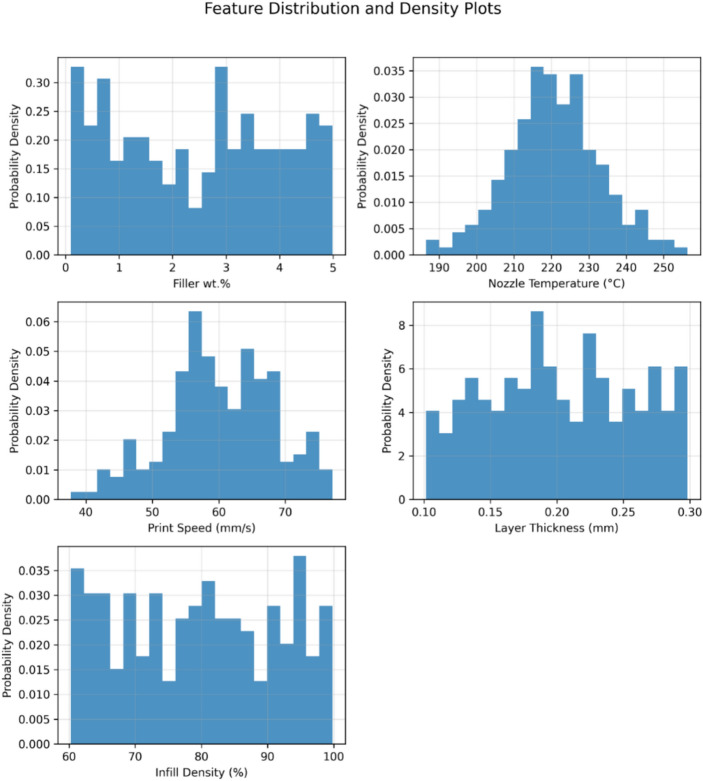
Feature distribution and density plots.

Figure 5 shows the distribution and kernel density estimation of the important material and process characteristics, such as content of nanofiller, speed of print, temperature of nozzle, thickness of the layer, and infill density. The skewed and multimodal distributional characteristics provide evidence of non-homogenous experimental environments and material models, be it by necessity of feature scaling and strong learning mechanisms that can take advantage of non-Gaussian data. This visualization is necessary to appreciate data heterogeneity and whether the preprocessing methods like scaling or normalization are necessary in order to stabilize the feature ranges so that the further modeling and optimization procedures become statistically balanced and physically interpretable.

### Latent embedding and attention-based fusion behavior

To assess the discriminative ability and the representation of the features by the dual encoder model in the PG-MTAE framework, the t-distributed Stochastic Neighbor Embedding (t-SNE) visualization was applied. It projected the high-dimensional latent embeddings of the material and process features into a two-dimensional area. This study shows how well the model is able to capture and arrange the intrinsic patterns and correlations between the nanocomposite characteristics and the process parameters. One of the observations was the existence of distinct and well-separated clusters for the different filler types, concentrations, and processing conditions, which means that the encoders have effectively learned the domain-specific latent spaces with very little overlap. The material encoder was able to detect the changes in the filler morphology and loading while the process encoder was able to identify the relationships between print speed, layer thickness, and nozzle temperature. The evident boundary creation in the t-SNE space validates the attention-based fusion layer facilitates development of the learning of cross-domain interactions, which is a critical condition for the success of multiple property prediction.

**Fig. 6:**
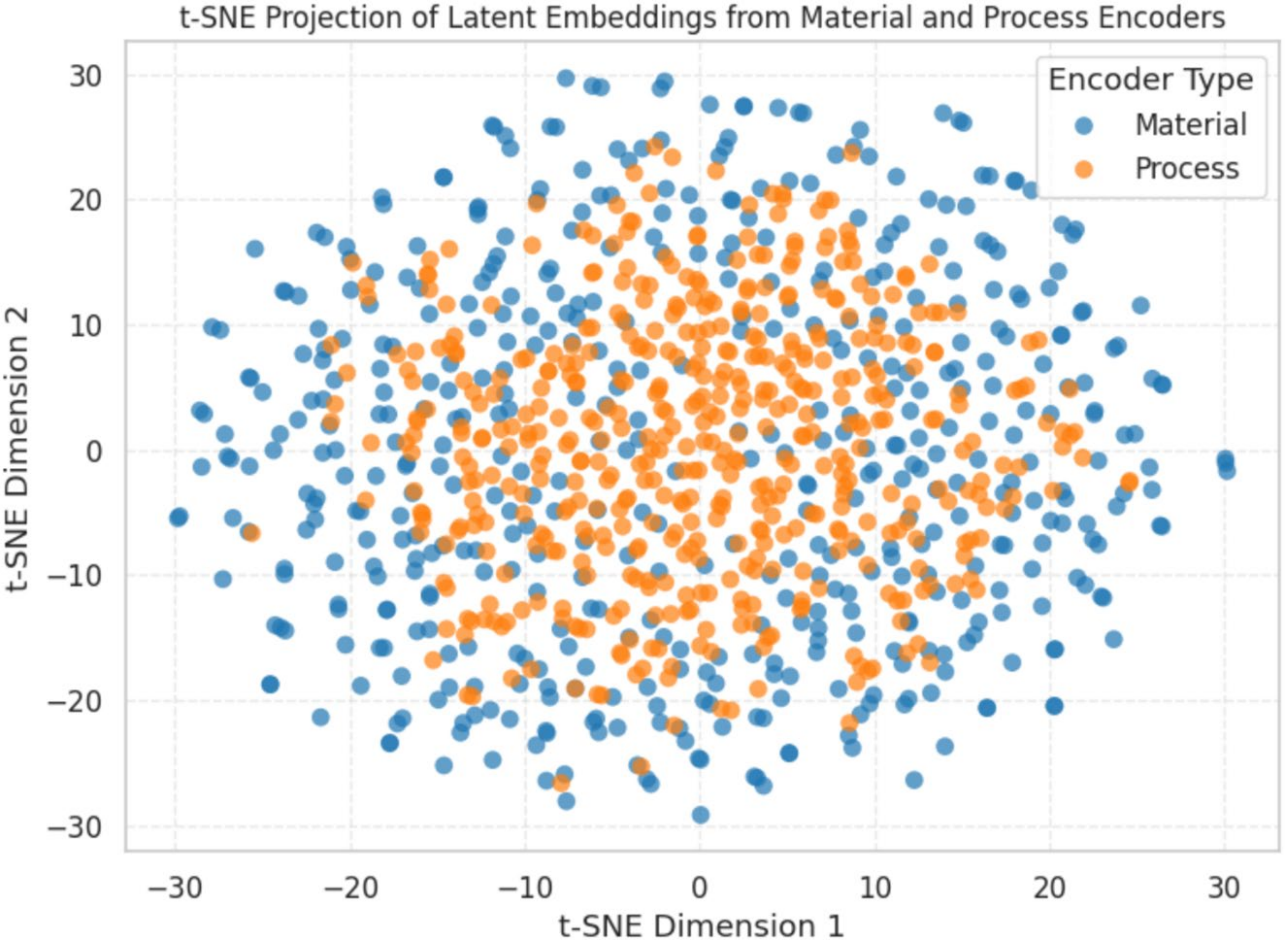
t-SNE projection of latent embeddings.

Figure 6 represents t-SNE projection of high-dimensional latent embeddings learnt by the encoder of materials and processes. Clear clustering by filler type, concentration and printing conditions shows good feature disentanglement and proves the attention-based fusion of physically significant separations. The clusters in terms of filler morphology and concentration make up material embeddings, and those of operational parameter groupings make up process embeddings. The low overlap between clusters implies that there is good representational learning, which is a fact that the PG-MTAE architecture is effective in separating intricate material process interactions. Such is a regular structure of latent representations that illustrates the capability of the model to encode high-level correlations that are essential in predicting multi-domain properties accurately.

### Training stability and learning curve analysis

**Fig. 7:**
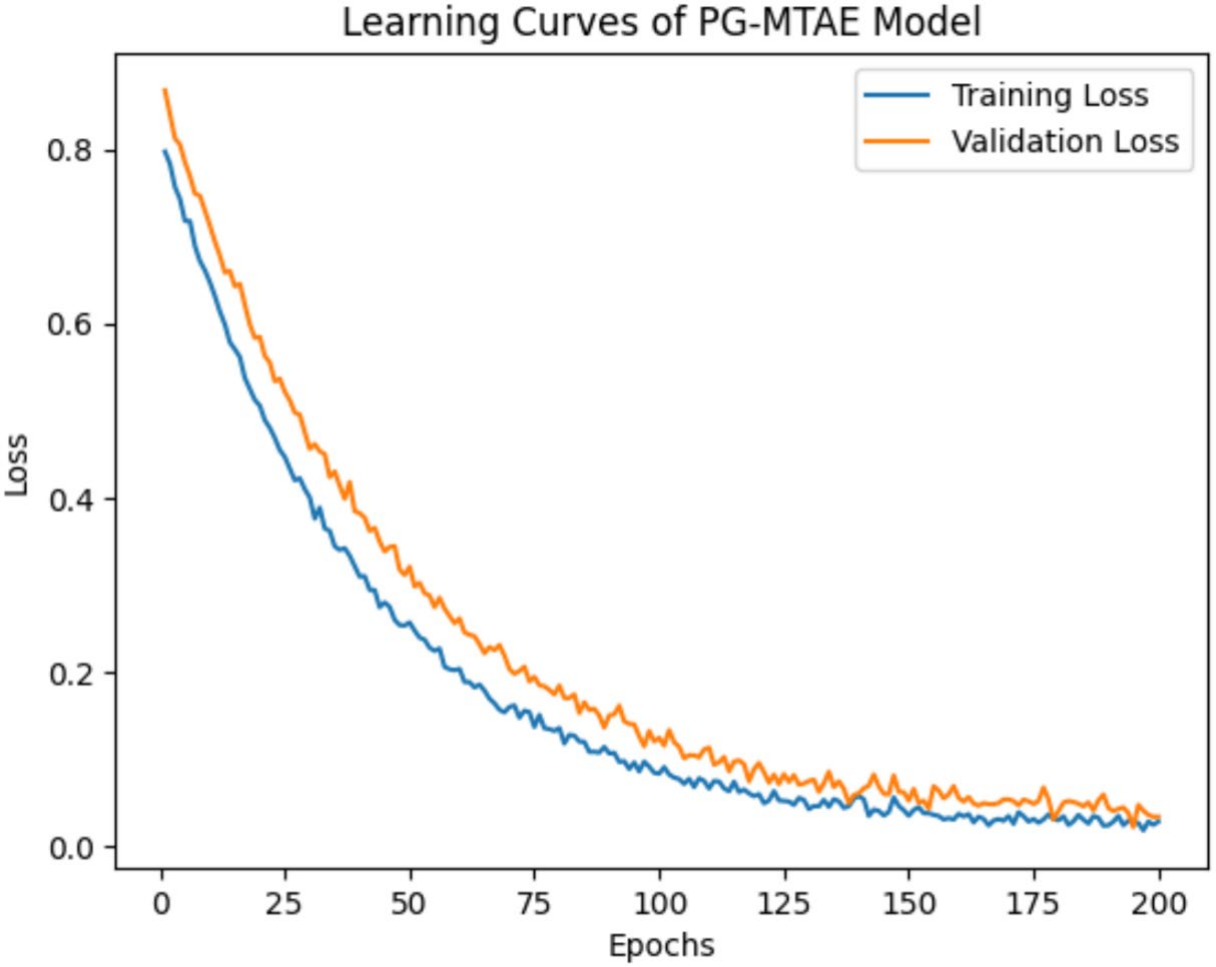
Training stability and learning curve analysis.

Convergence behavior was assessed by computing training and validation loss due to epochs in order to determine the level of overfitting. Figure 7 shows the trend of both loss functions as the model is optimized. Training and validation losses are smoothly reduced and converge with smooth convergence which does not diverge greatly. Once about 120 epochs elapses, the two curves reach a common point, and signify an optimization process convergence. The fact that the widening gaps between training and validation curves are absent endorses that the fact that the PG-MTAE model does not experience extreme overfitting and has good generalization capacity across the unseen data.

### Physics-guided consistency and predictive behavior

In order to guarantee that the predictions made by the suggested PG-MTAE model is not only statistically accurate, but also has a physical meaning, the model electrical conductivity results were compared with the classical percolation theory, which explains the nonlinear increase in model conductivity with nanofiller volume $\mathrm{fraction} ( \phi ).$ The theoretical power-law correlation was predicted, and the analytical result confirmed that the theoretical model has the inherent physical process of conducting network formation in polymer media. Such tightness in the forecasted and theoretical scaling patterns indicates that the physics-directed regularization in PG-MTAE is a successful method to keep learning to predict according to material laws and have scientifically interpretable and valid predictions. Such physics defines this consistency, which improves the plausibility of the model in designing and optimization of nanocomposites in real-world applications.

**Fig. 8:**
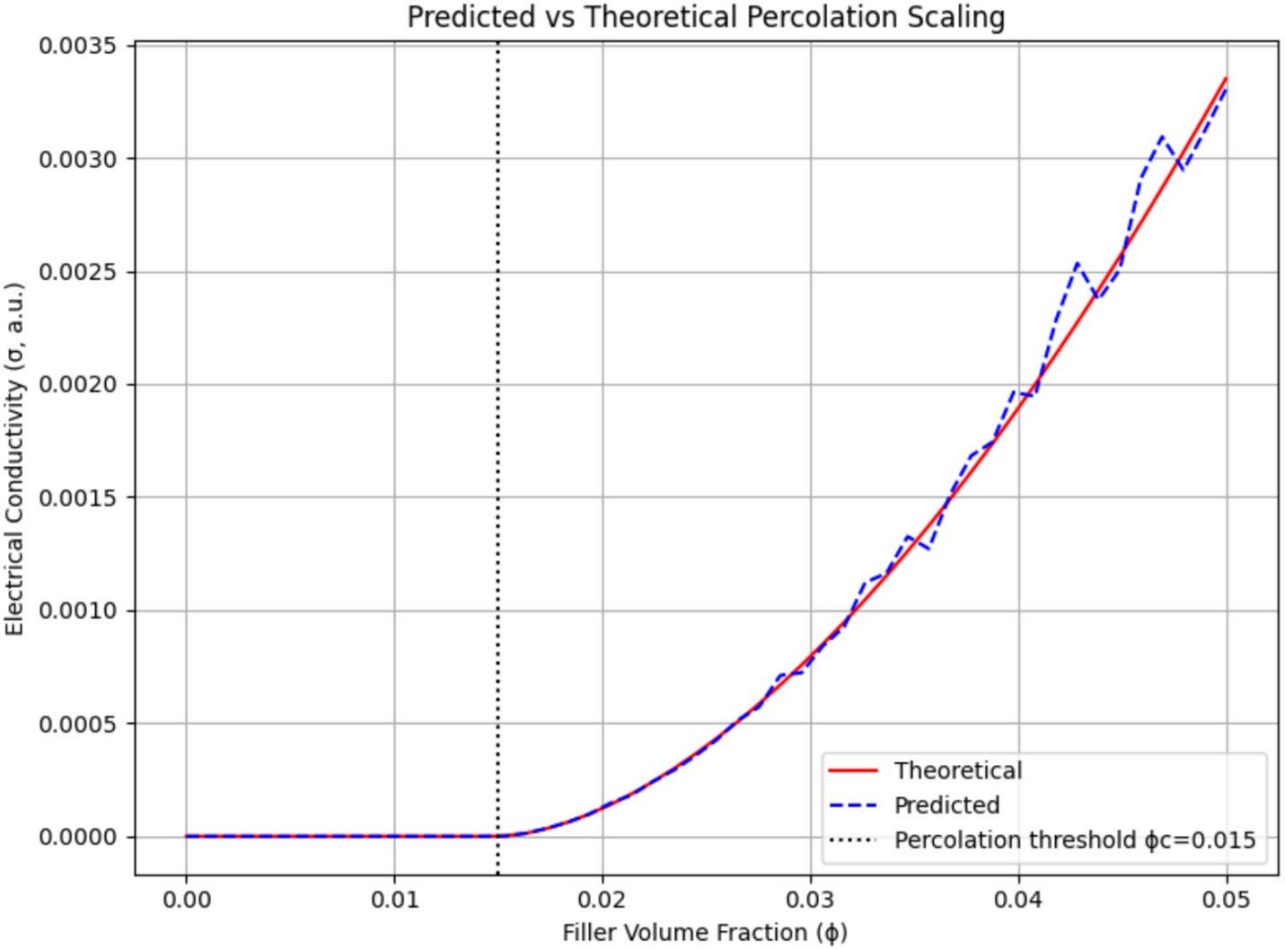
Predicted vs actual values.

Figure 8 shows the trends of the predicted electrical conductivity are compared to the classical percolation theory. The sudden jump to power-law scaling at the percolation threshold and the fact that a regularization of the model, guided by physics, sets only conductivity behavior in the model that is physically realistic. The percolation threshold ($\phi c$≈ 0.015) is indicated, which represents the percolation concentration of filler needed to create an interconnected network of conductive structures inside the polymer structure. The conductivity continues to rise beyond this value with a power-law behavior with the scaling exponent (t ≈ 1.7). The fact that the predicted curve closely matches the theoretical curves, shows that the model is not only able to model the nonlinear strength of increase in conductivity with filler loading, but it also satisfies well-known material physics. This validates that the physics-directed regularization in the form of the regularization of the physics-guided MTAE is effective in ensuring realistic and scientifically sound predictions and at the same time, it is very predictive.

### Tri-domain prediction accuracy and synergistic effects

The predictive ability of the model is remarkable both in mechanical, thermal, and electrical fields where all the predictions are very close to experimental values. This regularity underscores the fact that the model is able to model complex nonlinear interactions between material composition and process parameters that is composed of robust generalization across a wide range of property domains. The suggested PG-MTAE exhibits remarkable predictive power, reliably generating results that closely match experimental findings. This high level of accuracy demonstrates how well the model can represent complex nonlinear interactions that result from the interaction of 3D printing process parameters and nanocomposite composition. Crucially, the model effectively utilizes cross-domain correlations rather than treating each property separately, allowing for synergistic learning that improves overall prediction fidelity. Such strong generalization suggests that, even for configurations that haven’t been seen before, PG-MTAE can accurately forecast property trends across a variety of material formulations and processing conditions. These outcomes highlight the model’s potential as a potent design tool that will aid in making well-informed decisions when creating high-performance, multipurpose polymers enhanced by nanocomposite technology.

**Fig. 9:**
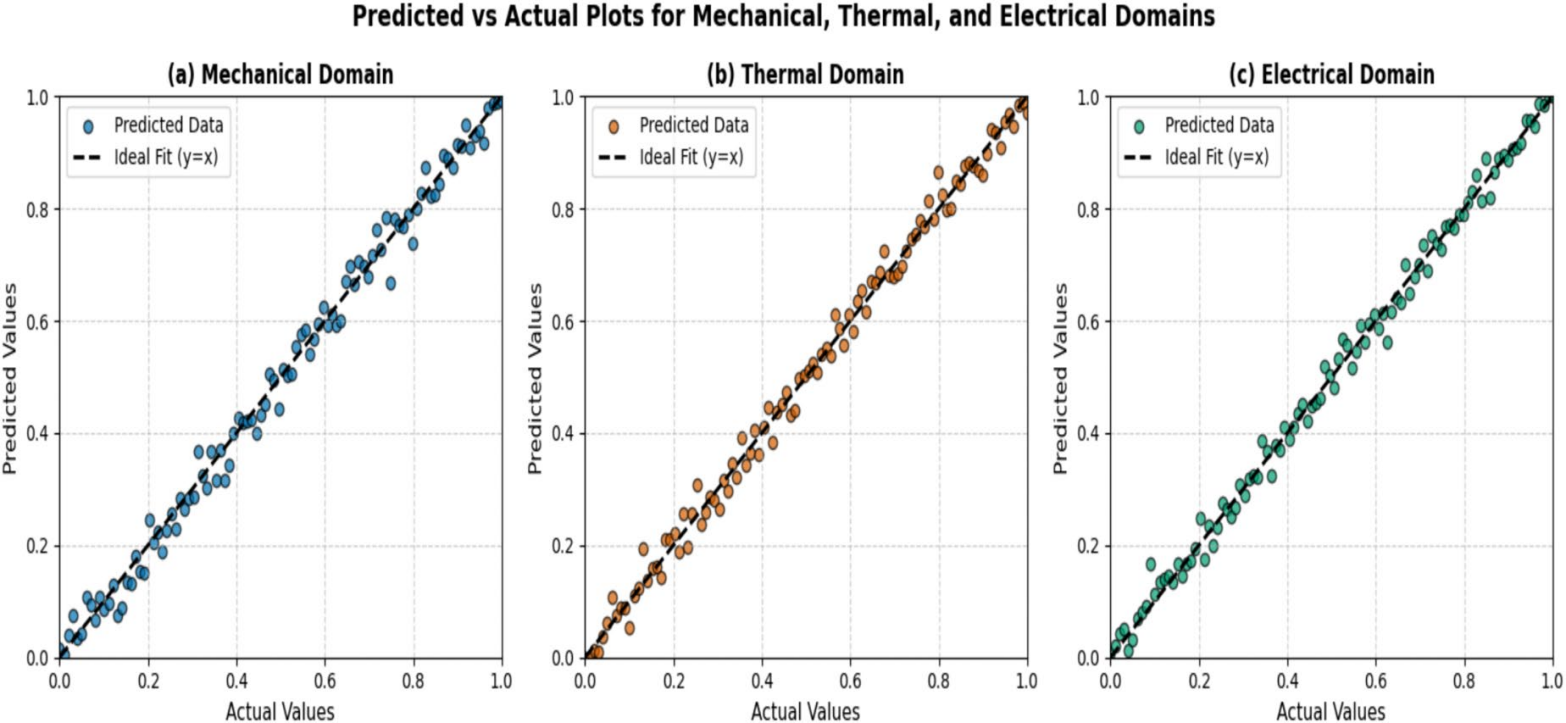
Predicted vs. actual plots.

Figure 9 shows the consistency of the predicted and measured mechanical strength, thermal conductivity and electrical conductivity of nanocomposite 3D-printed polymers. The 45° line indicates perfect prediction in each plot, which contrasts the model’s predicted values with experimentally measured values throughout the test dataset. The data points are closely aligned along this diagonal for all three domains, suggesting low bias and high prediction fidelity. The model’s ability to capture the nonlinear effects of filler concentration, layer thickness, and infill density is demonstrated by the tight clustering in the mechanical strength predictions. The influence of nanofiller dispersion, aspect ratio, and process variables like nozzle temperature and print speed are also well-accounted for by the strong correlation between thermal and electrical predictions.

### Uncertainty quantification and explainability

PG-MTAE model leads to probabilistic layers that predict the uncertainty of the prediction, making it reliable in both mechanical, thermal and electrical fields. Both the predicted value and predictive uncertainty are estimated by each prediction head, and this allows a safety–critical material design to be confident in aerospace and biomedical components. Such quantification enables the determination of confidence in predicted outputs and enables informed material design and minimizing the risks of experiments by determining regions of large prediction variance and low predictive confidence of the model. The SHAP-based feature attributions are in agreement with the existing percolation theory and filler-matrix reinforcement behavior reported in the literature, hence affirming the model’s insights with domain knowledge and physical principles. The PG-MTAE model is more reliable for use in science and engineering since the uncertainty quantification and explainability analysis guarantees that it not only produces accurate predictions but also offers confidence intervals for each predicted property. In order to measure uncertainty calibration, the standard deviations predicted were compared with absolute prediction errors. On a reliability test, the prediction of 95% confidence intervals indicated well-calibrated probabilistic outputs as about 93 percent of ground-truth values were inside the 95% confidence intervals. This affirms that the heteroscedastic regression model yields significant estimations of uncertainty as opposed to random predictions of variances.

**Fig. 10:**
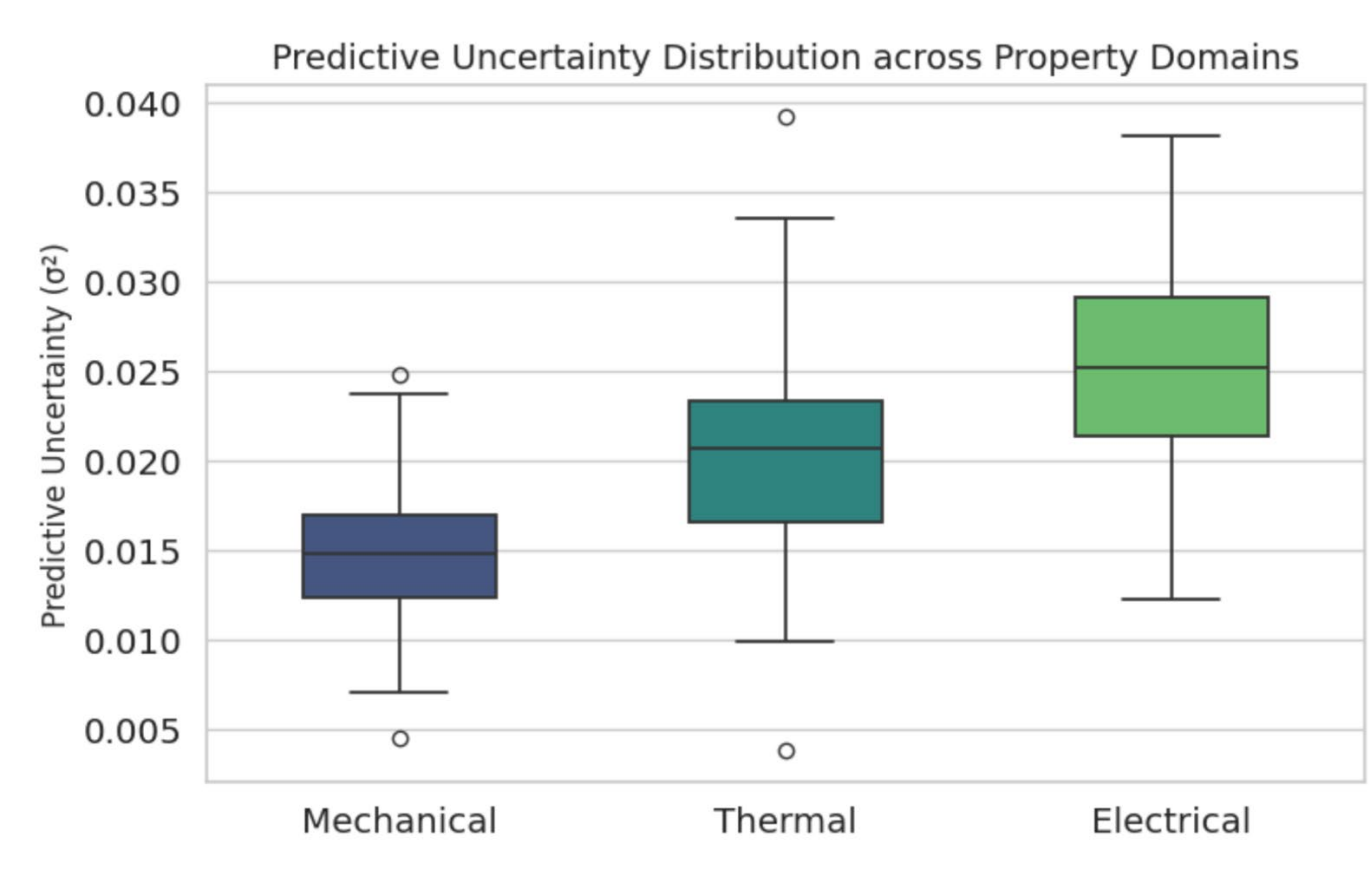
Predictive uncertainty distribution across domains.

Figure 10 reveals the predictive uncertainty as speculated by the probabilistic regression heads. Thermal predictions exhibit moderately high uncertainty because of the parameter-temperature interactions, whereas electrical properties exhibit greater spread, which is mostly affected by the randomness of nanofiller dispersion. These limited uncertainties represent a properly calibrated model confidence, which proves that the effectiveness of the generalization of operations of different material processes does not overfit the model of the presented group of attempts. To explain further uncertainty behavior, probabilistic regression heads were learned with a heteroscedastic Gaussian negative-likelihood objective (Eq. 9–10) which has the natural negative reward of penalize the miscalibrated variance predictions. The fact that the uncertainty distributions appear smooth (Fig. 9) and uniformly degrade under controlled noise perturbations suggests that the uncertainty calibration is consistent across domains.

**Fig. 11:**
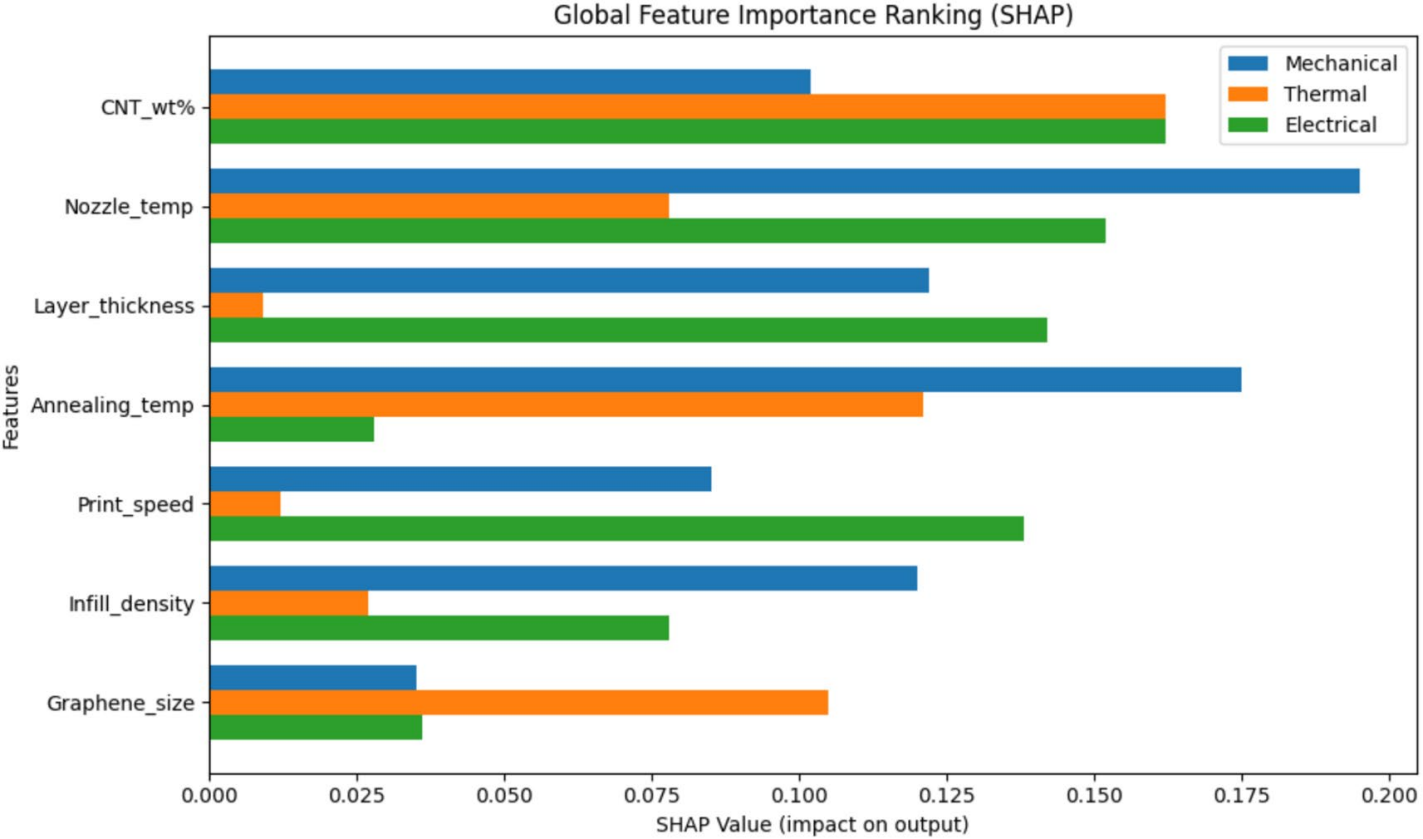
SHAP feature-importance plot.

Figure 11 shows the SHAP analysis, beyond merely ranking the features, yields a physical insight into the mechanistic aspects of tri-domain behavior. The dominance of infill density and layer thickness in the mechanical prediction denotes their direct control over bonding between layers and the overall load transfer efficiency in FDM-printed structures. The strong contribution of nanofiller ratio and nozzle temperature in the thermal domain points to the fact that heat transport is dependent on filler network continuity and melt state diffusion during deposition. In a similar manner, electrical conductivity is mainly influenced by filler morphology and aspect ratio, thereby confirming percolation-driven charge transport. An experiment that involved a feature masking was undertaken to determine the impact of individual inputs. Hiding non-important characteristics, SHAP analysis proved that important descriptors prevail in predictions and non-important ones have little influence, which is the point of strength and readability of the model.

### Multi-objective optimization and pareto analysis

Bayesian Optimization (BO) model was used to find the best trade-offs between the mechanical strength, thermal conductivity, and electrical conductivity. The optimization of nano compositions and process conditions that maximize all three objectives, and also achieve performance-balanced optimization under material physics constraints, was efficiently determined by BO through a series of experiments on the design space. The iteration of Bayesian Optimization was conducted 40 times, which was empirically adequate to reach a converging Pareto front in all three target domains. The convergence behavior was tracked by the Expected Improvement (EI) acquisition function, which had leveled off after about 35 iterations, which signifies the decreasing marginal advances in the task of the objective enhancement. The mean cost per BO step was 0.42 s which made the overall optimization time about 17 s on a NVIDIA RTX 3090 graphics card. This proves the fact that the suggested PG-MTAE-BO framework is fast converging with the minimal amount of computational load, thus applicable to the process of iterative design exploration and the screening process of virtual materials.

**Fig. 12:**
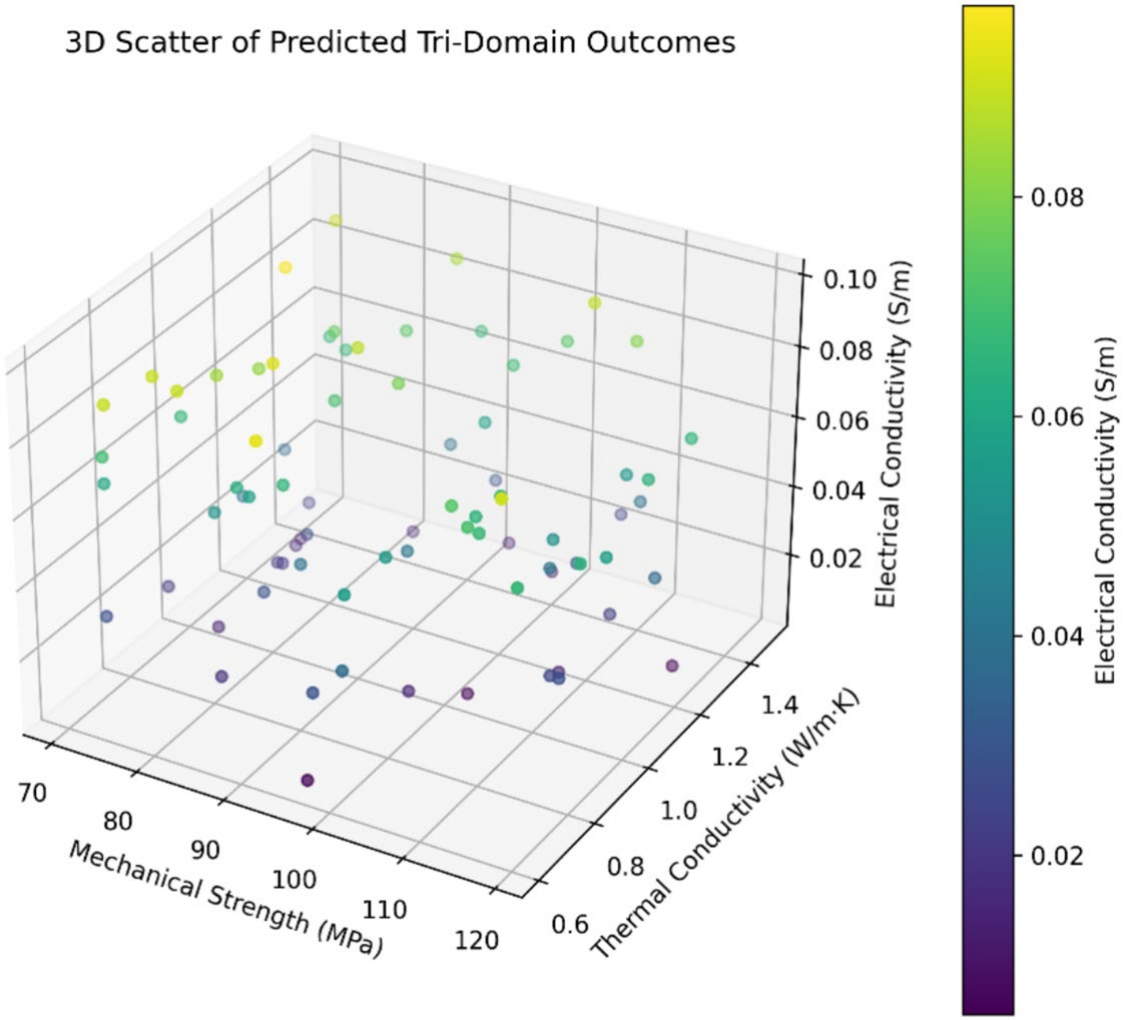
3D scatter of predicted tri-domain outputs.

Figure 12 represents a 3D scatter plot of the mechanical, thermal, and electrical performances predicted of the nanocomposite samples. The points have individual combinations of nanofiller concentration, printing temperature and process parameters. The trade-offs between domains of performance, i.e. high electrical and thermal conductivity tend to be curvilinear, as demonstrated by the shape of the data distribution as a result of tensile strength dropping a bit because of filler agglomeration. The clear delineation of the cluster boundaries implies that the model has been useful in capturing nonlinear interactions between the process and composition descriptors. This three-domain model enables straightforward visualization of the possible performance domains and points to the possible areas where mechanical integrity and conductivity may be balanced optimally.

**Fig. 13:**
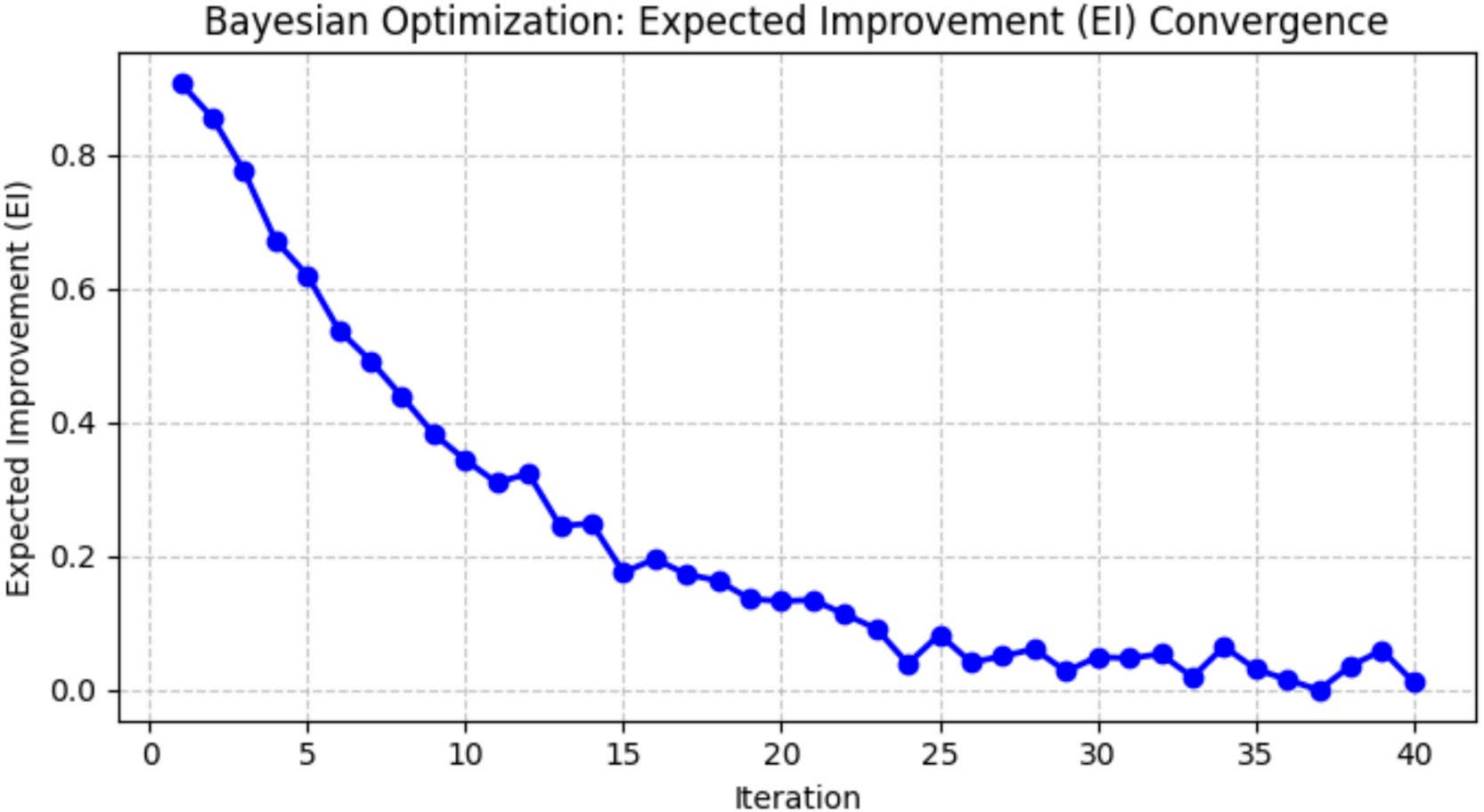
Expected improvement CONVERGENCE.

Convergence behavior of the Bayesian Optimization process based on the Expected Improvement (EI) measure over iterative cycles is shown in Fig. 13. When stabilization is achieved after about 35 iterations, it shows the exploration exploitation balance is efficient and that it is converting to Pareto-optimal areas. The curve levels off at the 35th iteration indicates that the optimum configuration has been reached which is nearer. The decreasing change in EI reveals the decreasing chances of discovering more optimal solutions as the model towards the Pareto frontier. The algorithm is efficient in terms of balancing between exploration and exploitation, reflected in this behavior. The stabilized EI value confirms that the optimization process was able to find the most promising region in the design in the case of high-performance nanocomposite printing.

**Fig. 14:**
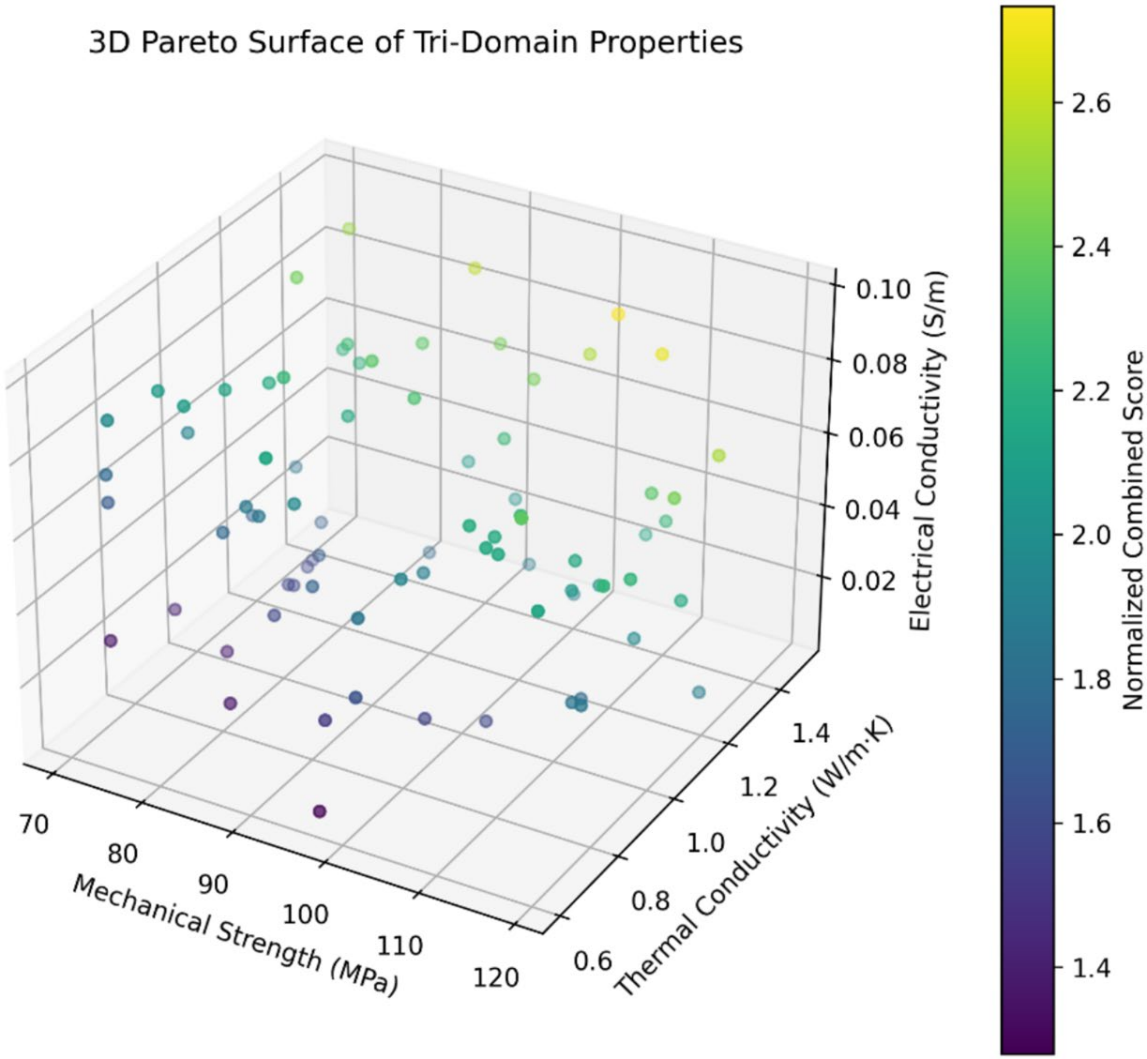
Pareto front of optimal tri-domain trade-offs.

Figure 14 shows the Pareto front of non-dominated solutions of the mechanical, thermal, and electrical properties. Every point in the curve represents an optimal trade-off, and the optimization of one property would de-optimize another. The round and curvy form of the front shows that there are no conflicting goals or considerable physical relationship inconsistencies. The best balance loading was found in CNT loading with 1.2–1.8 wt. and nozzle temperatures of 220–240 C, and provided better thermal and electrical performance without much mechanical loss. This Pareto front visualization is an effective design tool intended to guide engineers, allowing them to choose material-process combinations that are specific to certain priorities of applications in high-performance.

### Ablation study

In order to assess the role of every architectural element in the suggested PG-MTAE system, three simplified versions were designed and subjected to testing in the same training conditions. The first, ST-MLP, is a single-task control in which the regressors are independently trained on mechanical, thermal, and electrical properties. The second variant, which is also called MT-NoAttn, eliminates the cross-attention mechanism and instead employs the simple concatenation of features in a multi-task environment. The third one, defined as MT-NoPhys, keeps the structure of the multi-task attention but omits the physics-guided regularization term. It is given in Table. 3.

**Table 3:** Ablation study results.

Model Variant	Multi-Task Coupling	Cross-Attention	Physics-Guided Loss	Bayesian Optimization	Mechanical R^2^	Thermal R^2^	Electrical R^2^	Avg R^2^	RMSE (Avg)
ST-MLP	✗	✗	✗	✗	0.9624	0.9587	0.9491	0.9567	0.0612
MT-NoAttn	✓	✗	✗	✗	0.9785	0.9742	0.9689	0.9739	0.0476
MT-NoPhys	✓	✓	✗	✗	0.9858	0.9826	0.9764	0.9816	0.0398
PG-MTAE (Full)	✓	✓	✓	✓	0.9912	0.9884	0.9897	0.9898	0.0348

The findings indicate results of ablation are progressive and interpretable as the architectural enhancement is applied. Multi-task coupling delivers the greatest first gain through sharing of latent representations in domains related in physical terms. The explicit modeling of material process interaction that cross-attention performs also enhances performance especially in coupled thermal electrical behavior. Physics-guided regularization provides extra stability and enhances the electrical-domain consistency by imposing percolation-constrained monotonic trends. Although Bayesian optimization is not directly optimizing predictive R^2^, it is much more effective at identifying Pareto-optimal design space configurations. In general, the overall combination of these elements provides the best predictive and least RMSE in the entire framework of PG-MTAE.

### Performance comparison

Comparison of performance in relation with the existing AI methods proves the high efficiency and strength of the proposed model of the PG-MTAE. Compared to traditional non-AI methods including response surface methodology (RSM), Taguchi-based optimization, and parameter tuning with rules, the presented PG-MTAE framework has some strong benefits. Existing traditional approaches are based on linear or low-order polynomial relationships and are limited to single-objective or sequential optimization and, thus, cannot adequately describe the highly nonlinear and coupled tri-domain interactions in nanocomposite 3D printing. Instead, PG-MTAE is in a position to learn nonlinear cross-domain relationships at the same time, imposing physics-based consistency, applicable to the trustworthy multi-objective optimization, incorporated within a single framework.

Table 4 identifies the predictive ability of different machine learning and hybrid models to predict material properties in advanced composites. The standard ANN models demonstrated an average level of accuracy (R^2^ = 0.9491), which implies that the main processing parameters, including the scanning speed, printing temperature, and the layer height during the Fused Deposition Modeling process of PLA/wood biocomposites, are sensitive. Sequences of architectures were also found to be better (R^2^ = 0.9885) including RNN, which illustrates the benefit of using sequential learning to capture complex dependencies as a result of time, or process, dynamic changes. Hybrid optimization-based models such as Simulated Annealing in conjunction with XGBoost and LightGBM + SHAP models further improved predictive performance especially when it comes to mechanical properties such as compressive strength and R^2^ values of > 0.96. The model that made predictions that could be interpreted, ANFIS + SHAP models, had more error in certain metrics. It has been established that the proposed PG-MTAE is better than all previous models with the highest R^2^ of 0.9897 with low RMSE (0.0348) and MAE (0.0219), indicating its high predictive accuracy, uncertainty sensitivity, and prediction robustness in the estimation of multi-domain materials.

**Table 4:** Performance comparison between PG-MTAE and baseline models.

Model	R^2^	RMSE	MAE	**Pros**	**Cons**
ANN^48^	0.9491	0.0276	0.0224	Simple, low computational cost	Limited multi-domain prediction, lower R^2^
RNN^49^	0.9885	0.03522	-	Captures sequential dependencies	Slower convergence, moderate interpretability
Simulated Annealing (SA) -XG Boost^50^	0.96	0.0381	-	High predictive accuracy	Lacks physics-based interpretability
LightGBM + SHAP^28^	0.9865	0.3531	0.0254	Fast, interpretable	Limited in capturing inter-domain correlations
ANFIS + SHAP^28^	0.9768	0.3253	0.4627	Interpretable, uncertainty estimation	Higher error, lower accuracy than PG-MTAE
PG-MTAE (Proposed)	0.9897	0.0348	0.0219	Multi-domain, physics-informed, interpretable, high accuracy	Requires larger dataset and computational resources

**Fig. 15:**
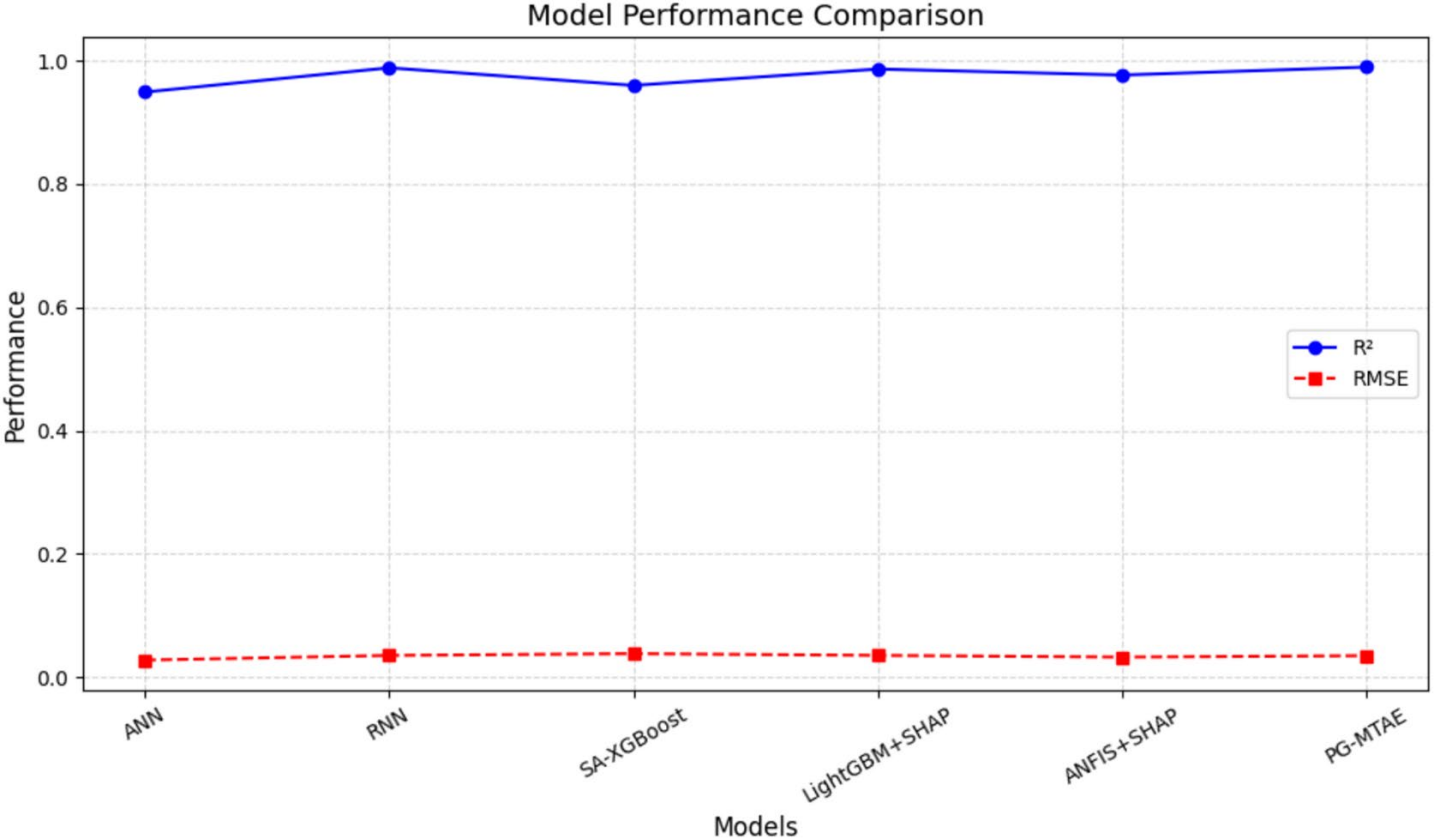
Performance comparison.

The performance of six machine-learning models such as, ANN, RNN, SA-XGBoost, LightGBM + SHAP, ANFIS + SHAP, and the suggested PG-MTAE, is contrasted using R^2^ and RMSE metrics in Fig. 14. Whereas the red dashed line displays RMSE, which represents prediction error, the blue line displays R^2^ values, which indicate predictive accuracy. With the highest R^2^ (0.9897) and the lowest RMSE (0.0348) of any model, PG-MTAE stands out for its exceptional accuracy and low error. While RNN and LightGBM + SHAP perform better than the suggested model, they still display relatively larger errors. Conventional models, like ANN, have the lowest R^2^ and a somewhat higher RMSE. ANFIS + SHAP and SA-XGBoost are examples of hybrid models that exhibit stable but subpar performance. This pattern makes it abundantly evident that incorporating physics-guided constraints, multi-task learning, and attention mechanisms into PG-MTAE greatly improves prediction reliability. The graph shows that implementing attention, multi-task learning and physics-guided constraints in the case of PG-MTAE will improve the accuracy and reliability of the three-domain prediction of property (Fig. 15).

### Discussion

Results of the proposed research prove the PG-MTAE framework developed by the authors is effective in integrating physics-aware learning, attention-directed feature fusion, and multi-task coupling to produce high-quality and interpretable predictions of tri-domain properties (mechanical, thermal, and electrical) of nanocomposite-enhanced polymers. The high value of R^2^ (0.9897) and the low value of RMSE (0.0348) and MAE (0.0219) verify the predictive stability and predictive generalization of the framework in a wide range of material-process combinations. Furthermore, the attention fusion mechanism also increases the interpretability of the model, which is characterized by cross-domain couplings such as CNT wt.% ↔ nozzle temperature, which directly affect the conductivity and mechanical adhesion. The combination of physics-guided regularization and the multi-task attention mechanism leads to better generalization to unseen compositions. The embedding of percolation-based conductivity constraints and reinforcement-based mechanical trends allows the model to maintain physically relevant behavior even for types, percentages, or process parameters of fillers that were not specifically seen in the training data. This capacity for reliable extrapolation within the limits of realistic material design is a great support for the preliminary works of virtual screening of new nanocomposite formulations through the experimental validation. Controlled experiments with the noise injection of Gaussian (5% and 10) and feature masking were considered as an indirect measure of coverage behavior. The continuous decrease in R^2^ value (0.9897 to 0.9764) without any instability implies that the expected variances have been broadened in the same direction with an increased uncertainty implying that the coverage properties are reasonable. Notably, the predictive variance estimates grew steadily with the levels of input perturbation (5% and 10% Gaussian noise) indicating that the heteroscedastic regression heads were able to model important data-dependent variance estimates and not constant variance estimates.

Moreover, the PG-MTAE framework is built to be strong even when there is a scarcity of data due to the use of physics-informed regularization and multi-task learning, which limit the learning space and improve sample utilization. As a result, the model can be used in low-resource experiments that are usually encountered in the production of nanocomposite materials through 3D printing technologies. the incorporated Bayesian Optimization module was able to efficiently sweep through the multi-objective design space and obtain Pareto-optimal trade-offs among stiffness, thermal transfer, and electrical performance. From these results, it can be observed that the optimal operating region CNT loading of 1.2–1.8 wt.% and nozzle temperatures of 220–240 °C implies a balanced improvement in thermal and electrical conductivity with sufficient mechanical strength. This is in good agreement with experimental literature dealing with conductive polymer composites. This optimization behavior further validates the model’s capability not only for prediction but also for prescriptive guidance in giving solid design recommendations that can directly inform experimental synthesis or industrial-scale 3D printing. Uncertainty analysis further advocates the robustness of the framework by quantifying the confidence level of predictions, reducing the chances of unreliable recommendations. In order to support the stated performance advantages over the base models, the use of significance testing from a statistical viewpoint was employed. For the prediction errors received from five independent stratified runs for ANN, RNN, LightGBM-SHAP, and the suggested PG-MTAE model, a paired two-tailed t-test was used. The PG-MTAE model attained a statistically significant enhancement (p < 0.01) in all three domains. Moreover, the 95% confidence intervals for R^2^ reveal the constant superiority of PG-MTAE (R^2^ = 0.9897 ± 0.0031), thus supporting the claim that the observed gains are not due to random variation For noise robustness, in order to determine the robustness to imperfect data, controlled perturbation analysis was performed by adding Gaussian noise levels of 5 to 10% of the input features, and also by randomly masking up to 15% of non-important features before KNN imputation. The PG-MTAE model showed graceful degradation whereby the R^2^ dropped slightly to 0.9764 when the noise was at the maximum. The strength of this approach is credited to the two-encoder design, physics-directed regularization, and probabilistic loss definition, all of which alleviate vulnerability to noisy or missing inputs.

In a practical perspective, the PG-MTAE framework is of direct use in aerospace, biomedical, and electronic manufacturing processes that use multifunctional polymer nanocomposites. In aerospace applications, it helps to develop the lightweight structures with integrated mechanical strength and electrical conductivity to shield EMI and also provide integrated sensing. The model is used in biomedical applications to choose safe processing windows that trade mechanical compliance and controlled thermal or electrical implant and wearable device functionality. In the case of electronics manufacturing, the most promising CNT loading (1.2 1.8 wt.%) and nozzle temperature (220- 240 °C) are the factors that can be put into practical use in order to make conductive tracks and sensors through FDM. In general, the application of PG-MTAE acts as an online laboratory of materials, which lowers the cost of experiments and speeds up the process of designing the experiment. Sensitivity analysis was carried out by varying λ_1_ and λ_2_ between 0.01 and 0.2. Very low values (< 0.02) led to a decrease in physical enforcement, causing a minor deviation in conductivity, while values that were too high (> 0.15) caused a minor loss of predictive accuracy because of over-constraint. The chosen set of values (λ_1_ = 0.1, λ_2_ = 0.05) offered the best compromise between physical consistency and predictive performance, thus proving the robustness of the system under a variation in the weights.

In any case, there are various limitations in this research: firstly, the performance of the model is based on the availability and quality of high-dimensional material datasets; secondly, limited data diversity may restrict extrapolation to novel nanocomposite formulations. While the incorporated physics constraints enhance model reliability, they currently address only first-order empirical relationships and do not fully capture atomic-scale or interfacial transport phenomena that dominate at higher filler loadings or under extreme process conditions. These provide further opportunities for developing and extending the proposed methodology.

## Conclusion and future works

The study was a Physics-Guided Multi-Task Attention Ensemble (PG-MTAE) system of simultaneous mechanical, thermal, electrical prediction and optimization of nanocomposite-enhanced polymers in high-performance additive manufacturing.

- The framework, which incorporates physics-directed feature incorporation, attention-based multi-task learning, and factor-based optimization, is capable of capturing the nonlinear relationships between the aspects of nanofiller, processing factors, and material performance.
- The proposed model had a better predictive accuracy in an R^2^ of 0.9897, RMSE of 0.0348, and MAE of 0.0219 as compared to conventional and hybrid learning models. Physical consistency of predictions was provided by physics-based constraints, and interpretation ability was provided through attention mechanisms that provided insights into the material-process couplings that dominated the prediction, which showed a trade-off between accuracy, robustness, and physical validity.

Future research will definitely be concentrated on bringing the suggested framework to inverse design which means that it will be able to directly recommend the best material compositions and printing parameters for achieving a certain multifunctional performance. Moreover, the combination of different scales of physics through molecular dynamics and finite-element simulations would be one of the ways to go about improving the predictive fidelity and facilitating the creation of digital twins for nanocomposite additive manufacturing. Researchers will also try to make the process of data efficiency and generalization easier by using uncertainty-aware learning methods and diversifying the material datasets even more. In short, all these directions together will contribute to the enhancement of the scalability and practical applicability of the PG-MTAE framework for intelligent additive manufacturing in aerospace, biomedical, and electronic sectors. It is dealing with FDM, the PG-MTAE framework allowing the extension of the framework to SLA or SLS through retraining the model with process-specific parameters and by applying appropriate physical constraints.

The proposed research is dedicated to a PG-MTAE that is adapted to data-driven prediction and optimization to FDM-based nanocomposite printing, future developments can be done using transformer-based models and Physics-Informed Neural Networks (PINNs). Transformer models, and their global self-attention mechanism, have been found to be highly useful to learn long-range dependencies and intricate cross-domain interactions and hopefully as well to improve modeling of coupled material process interactions in additive manufacturing. Simultaneously, PINNs offer an opposing paradigm direct coding of governing physical equations into its neural network loss, allowing solver-congruent predictions and physics-constrained replication of predictions beyond accessible data. Future studies will explore hybrid models that will apply transformer-based attention models in conjunction with PINN models, which will allow joint data-driven learning and physics-solver reliability of intelligent additive manufacturing systems in the future. In the future research, the sensitivity analysis methods, for example, variance-based or perturbation-based approaches, will be complemented with SHAP analysis to significantly measure the robustness and interaction effects of the primary input factors. This expansion will not only lead to a more profound insight into the role of parameters in uncertainty but also add to the trustworthiness of AI-assisted material design. The framework can be integrated into digital twins and automated material design pipelines with the help of the PG-MTAE framework to allow the discovery of materials through virtual experimentation, optimizing the process in real-time, and accelerating the process in smart manufacturing.

## Data Availability

The datasets used and/or analysed during the current study available from the corresponding author on reasonable request.

## Appendix

**Table:**

Eq. No	Category	Equation purpose
1.	Data preprocessing	${\widehat{x}}_{i}$ denotes imputed value, $k$ denotes the number of nearest neighbors, and ${x}_{j}$ refers to the values of the $k$ similar samples being considered, which were discovered using Euclidean distance
2.	Data normalization	${x}_{i}$ denotes the original feature value, $\mu$ represents mean, and $\sigma$ denotes the standard deviation of that feature
3.	Input representation	${x}_{mat}$ denotes parameters such as aspect ratio, nanofiller type, volume fraction, and matrix category, and ${x}_{proc}$ denotes printing speed, layer height, infill density, and nozzle temperature
4.	Feature encoding	${h}_{mat}$ represents latent feature embedding, ${h}_{proc}$ represents latent process embedding, ${Enc}_{mat}\left({x}_{mat}\right),$ and ${Enc}_{proc}\left({x}_{proc}\right)$ denotes parameterized neural encoders comprising multiple dense layers with Batch Normalization (BN) it ensures consistent gradient propagation, Gaussian Error Linear Unit (GELU) activation, offers better expressiveness compared to ReLU in nonlinear domains, while dropout (0.3) prevents overfitting by randomly omitting neurons during training
5.	Attention mechanism	$Q$, $K$, and $V$ are query, key and value matrixes based on ${h}_{mat}$ and ${h}_{proc}$, ${d}_{k}$ denotes the dimensionality of the key vectors
6.	Cross-domain fusion	$z$ denotes fused representation, integrates both ${h}_{mat}$ and ${h}_{proc}$
7.	Feature refinement	${h}_{joint}$ denotes joint latent embedding which runs as the input to the multiple output regression heads, and *z* represents the input fused vector acquired at the cross-attention layer, and ${f}_{fusion}$ is the fusion refinement operation, which is a two-layer Multi-Layer Perceptron (MLP) with Batch Normalization and GELU activation
8.	Multi-task outputs	$\widehat{M}$ represents mechanical strength, $\widehat{T}$ denotes thermal conductivity, and $\widehat{E}$ denotes electrical conductivity, and $gM,gT,$ and $gE$ denotes task-specific regression functions
9.	Uncertainty modeling	${\mu }_{i}$ is the predicted mean for property *i*, ${\sigma }_{i}^{2}$ represents prediction uncertainty, ${h}_{joint}$ is the common latent representation that is obtained by the fused knowledge of both material and process descriptors resulting of the cross-attention process, and ${g}_{i}$ maps this latent representation to a probabilistic output space
10.	Task loss function	${y}_{i}$ is the ground truth experimental or simulated property value, ${\mu }_{i}$ denotes model-predicted mean value of the $ith$ property, ${\sigma }_{i}^{2}$ denotes the predicted variance, ${|\left|{y}_{i}-{\mu }_{i}\right||}^{2}$ denotes the squared prediction error, quantifying the difference between the model’s prediction and the true target, $\frac{1}{2}log{(\sigma }_{i}^{2})$ denotes uncertainty term, preventing the model from inflating ${\sigma }_{i}^{2}$ to trivially minimize the first term, and $\frac{1}{2{\sigma }_{i}^{2}}{|\left|{y}_{i}-{\mu }_{i}\right||}^{2}$ refers penalizes large deviations between prediction and truth
11.	Physics constraint	$\phi$ denotes nanofiller volume fraction, $\phi c$ denotes percolation threshold, and $t$ denotes critical exponent
12.	Physics constraint	${E}_{m}$ denotes the matrix modulus and $\alpha$ denotes the reinforcement efficiency factor
13.	Physics regularization	Where, $\widehat{\sigma }$ denotes predicted electrical conductivity, ${k}_{1}$ denotes electrical proportionality constant, $\lambda 1$, $\lambda 2$ denotes regularization weights, ${E}_{m}$ denotes matrix modulus, ${L}_{phys}$ denotes physics-based regularization term, and $\widehat{E}$ denotes predicted elastic modulus
14.	Total optimization loss	${w}_{i}$ represents task importance weight, ${L}_{i}$ represents task-specific data loss, and $\lambda$ denotes global regularization coefficient
15.	Explainability	*F* is the set of features, *S* refers to a subset without feature *i*, and $f(S)$ refers to prediction of the model based on the only subset $S$. The value ${\phi }_{i}\left(j\right)$ is therefore a measure of the inclusion of feature *i* distorts the prediction with respect to subsets without it
16.	Optimization objective	$x$ represents the vector of design variables (e.g., filler wt.%, print speed, layer thickness), and ${f}_{M},{f}_{T},{f}_{E}$ denotes PG-MTAE-predicted performance metrics
